# Natural Cyclic Peptides: Synthetic Strategies and Biomedical Applications

**DOI:** 10.3390/biomedicines13010240

**Published:** 2025-01-20

**Authors:** Devan Buchanan, Shogo Mori, Ahmed Chadli, Siva S. Panda

**Affiliations:** 1 Department of Chemistry and Biochemistry, Augusta University, Augusta, GA 30912, USA; dbuchan1@augusta.edu (D.B.); smori@augusta.edu (S.M.); 2 Georgia Cancer Center, Augusta University, Augusta, GA 30912, USA; achadli@augusta.edu; 3 Department of Biochemistry and Molecular Biology, Augusta University, Augusta, GA 30912, USA

**Keywords:** synthesis, natural products, cyclic peptides, biological properties

## Abstract

Natural cyclic peptides, a diverse class of bioactive compounds, have been isolated from various natural sources and are renowned for their extensive structural variability and broad spectrum of medicinal properties. Over 40 cyclic peptides or their derivatives are currently approved as medicines, underscoring their significant therapeutic potential. These compounds are employed in diverse roles, including antibiotics, antifungals, antiparasitics, immune modulators, and anti-inflammatory agents. Their unique ability to combine high specificity with desirable pharmacokinetic properties makes them valuable tools in addressing unmet medical needs, such as combating drug-resistant pathogens and targeting challenging biological pathways. Due to the typically low concentrations of cyclic peptides in nature, effective synthetic strategies are indispensable for their acquisition, characterization, and biological evaluation. Cyclization, a critical step in their synthesis, enhances metabolic stability, bioavailability, and receptor binding affinity. Advances in synthetic methodologies—such as solid-phase peptide synthesis (SPPS), chemoenzymatic approaches, and orthogonal protection strategies—have transformed cyclic peptide production, enabling greater structural complexity and precision. This review compiles recent progress in the total synthesis and biological evaluation of natural cyclic peptides from 2017 onward, categorized by cyclization strategies: head-to-tail; head-to-side-chain; tail-to-side-chain; and side-chain-to-side-chain strategies. Each account includes retrosynthetic analyses, synthetic advancements, and biological data to illustrate their therapeutic relevance and innovative methodologies. Looking ahead, the future of cyclic peptides in drug discovery is bright. Emerging trends, including integrating computational tools for rational design, novel cyclization techniques to improve pharmacokinetic profiles, and interdisciplinary collaboration among chemists, biologists, and computational scientists, promise to expand the scope of cyclic peptide-based therapeutics. These advancements can potentially address complex diseases and advance the broader field of biological drug development.

## 1. Introduction

Natural cyclic peptides, isolated from various natural sources (i.e., bacteria, fungi, plants, and marine species), have emerged as an interesting biomolecule class with remarkable diversity in shape, size, and chemical composition [1]. Cyclic peptides exhibit a wide range of therapeutic benefits for medicinal purposes [2], as over 40 cyclic peptides are currently approved and used as clinical therapeutics [3,4]. Two decades of research have highlighted their ability to inhibit enzymes, disrupt protein–protein interactions, modulate receptor signaling, and regulate immune responses [5,6,7,8,9,10,11,12,13,14]. Therefore, pharmaceutical advancement and medical research have proven them useful as antibiotics, antifungals, antihypertensives, chemotherapy and anti-autoimmune agents, antidiabetic and weight loss drugs, and anti-inflammatory and pain relievers [15,16,17,18]. Cyclic peptides are also valuable as drug discovery tools as molecular probes for identifying protein functions, disease mechanisms, or therapeutic targets.

The biological relevance of cyclic peptides is due to their ability to adopt rigid three-dimensional (3D) shapes and their limited conformational freedom [19]. Compared to linear peptides, cyclic peptides experience conformational stability from cyclization, resulting in definitive three-dimensional shapes with high surface areas. This allows for low-energy interaction barriers with target proteins and increased target protein binding affinity, increasing target specificity [20]. The constraint thus invoked by cyclization reduces nonspecific interactions by having limited conformational freedom and allows for the evasion of protease catalytic sites, extending their cellular half-lives. [21,22]. Collectively, cyclization enhances a peptide’s specificity and narrows the range of possible interactions, which is an attractive property for pharmaceutical development.

The synthesis of natural cyclic peptides is also much different in terms of challenges than linear peptides. From a synthetic standpoint, cyclization of linear peptides is often the most challenging step because cyclization is highly dependent on kinetic and thermodynamic factors. Thermodynamically, macrocyclization is not favored at equilibrium due to entropy losses from decreased rotational freedom and the high energy barriers associated with amide bonds. To overcome this, macrocyclizations must be performed under kinetic conditions, such as low concentrations (1 mM to 5 mM) and low, ambient, or mild temperatures. The reaction conditions and the reactivity of the termini involved control the kinetics of cyclization. For example, an efficacious cyclization resulting in an amide bond requires a sterically unhindered amine nucleophile and an activated carboxylic acid by a coupling agent for enhanced electrophilicity [23,24].

Natural cyclic peptides exhibit high complexity and a range of structural variability. To harness this specificity, many cyclization strategies have been developed based on the location of each reacting termini using common peptide terminology. Peptide cyclization can occur at the C-terminus (head), the N-terminus (tail, NH_2_, or OH), or the R-group of an amino acid residue (side chain). Four cyclization orientations are, therefore, possible: head-to-tail, head-to-side-chain, tail-to-side-chain, and side-chain-to-side-chain. Head-to-tail cyclization is the most common, resulting in a complete ring closure between the N-terminal amine and C-terminal carboxylic acid. Head-to-side-chain cyclization involves a nucleophilic side-chain bonding to the C-terminal head. Tail-to-side-chain cyclization links the N-terminus to an electrophilic side chain. Finally, side-chain-to-side-chain cyclization links R-groups of adjacent amino acid residues, such as forming a disulfide linkage via Cys oxidation. These cyclization strategies are chosen based on the target structure, the synthetic route, and the reaction conditions [25,26,27].

**Scheme 1:**
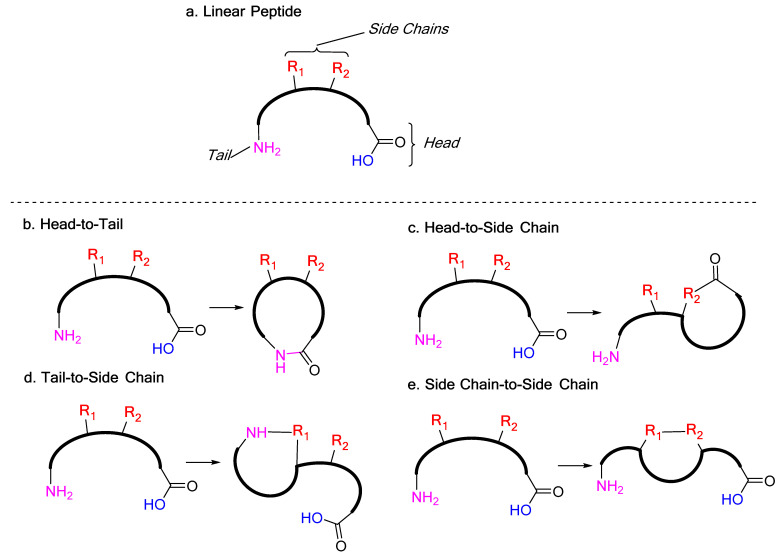
The four possible macrocyclization strategies for the synthesis of cyclic peptides. (**a**) Hypothetical cyclic peptide with nucleophilic head (NH_2_), electrophilic tail (COOH), an electrophilic side chain (R_1_), and a nucleophilic side chain (R_2_). (**b**) Head-to-tail cyclization. (**c**) Head-to-side-chain cyclization. (**d**) Tail-to-side-chain cyclization. (**e**) Side-chain-to-side-chain cyclization.

This review will present a comprehensive overview of the reported total syntheses of natural cyclic peptides, focusing on the cyclization approach and biological data performed since 2017. We have compiled case-specific total syntheses and approaches to achieve natural cyclic peptides, including partial retrosynthetic analyses and the biological data reported. Our review categorizes each synthetic report based on the abovementioned cyclization (Scheme 1) connection used. We aim to advance our understanding of natural cyclic peptides and their development as valuable therapeutics. We hope to provide synthetic insight and biological knowledge in order to welcome new ideas and perspectives in the intriguing field of study.

## 2. Head-to-Tail Cyclization

**Figure 1:**
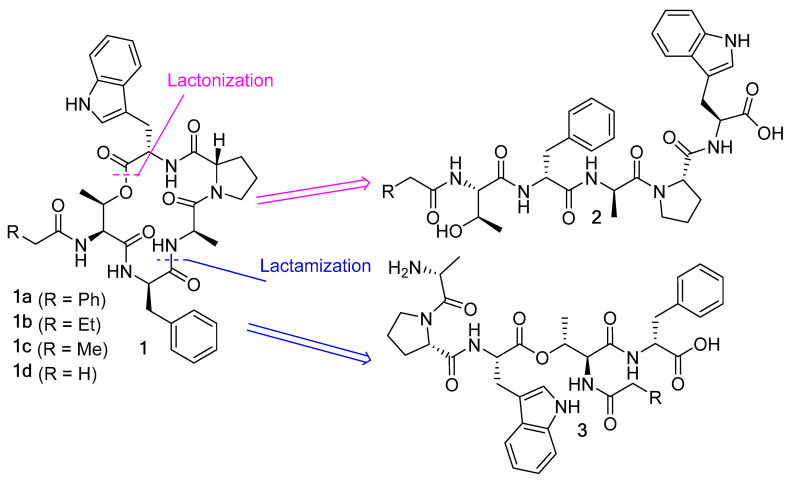
Retrosynthetic analysis of compounds **1a**–**d**. Macrolactamization versus macrolactonization.

**Figure 2:**
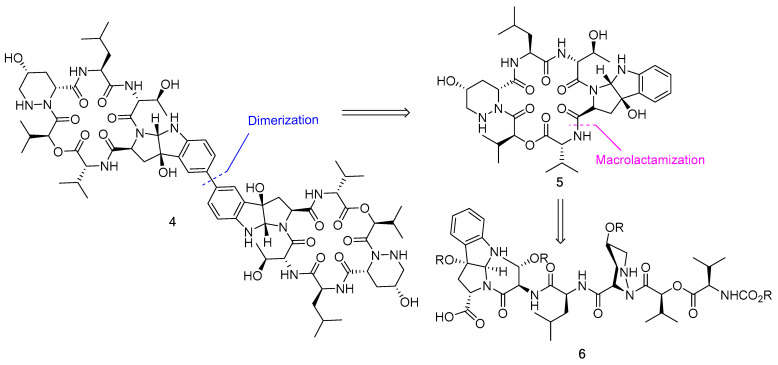
Retrosynthetic analysis of **4**.

Lu and Bately explored the total synthesis of chaiyaphumines A–D (**1a**–**d**, Figure 1), comparing the approach of head-to-side-chain macrolactonization (esterification) versus head-to-tail macrolactamization (amidation) and determining which is most effective [28]. Chaiyaphumine A (**1a**) has shown potential as an antiparasitic agent, with reported activity against *Plasmodium falciparum* (IC_50_ of 0.61 μM) [29], which causes malaria, and *Trypanosoma brucei rhodesiense* (IC_50_ of 5.11 μM) [30], responsible for causing East African sleeping sickness. The chaiyaphumines all have the same amino acids: l-Trp; l-Pro; d-Ala; d-Phe; and l-Thr, but differ in their side chain acylations (R-groups, Figure 1), which were accomplished after cyclization. The linear precursor was achieved through solid-phase peptide synthesis (SPPS) using hexafluorophosphate azabenzotriazole tetramethyl uronium (HATU) on a chlorotrityl resin. Head-to-side-chain macrolactonization was accomplished using 2-methyl-6-nitrobenzoic anhydride (MNBA), *N*,*N*-dimethylaminopyridine (DMAP), and Hunig’s base (*N*,*N*-diisopropylethylamine, DIPEA). No yield was observed unless dysprosium triflate (Dy(Otf)_3_, 30 mol%) was added to the reaction. The concentration of this reaction was also explored for optimization, reporting ideal concentrations of roughly 20 mM, resulting in isolated yields of up to 51% after purification. NMR characterizations showed some epimerization at the l-Trp residue. Synthesis of **1a**–**d** through head-to-tail macrolactamization was more efficient in the presence of 1-Ethyl-3-(3-dimethylaminopropyl)carbodiimide (EDCI), Hydroxybenzotriazole (HOBt), and Hunig’s base refluxing at a ~2 mM. The optimized conditions for macrolactamization reported yields up to 73%, and the addition of Dy(Otf)_3_ did not affect yields. The residues utilized under the latter conditions were assumed to have the lowest strain and superior kinetics compared to the macrolactonization location. To complete the synthesis of **1a**–**d**, an acylation reaction was placed at the free amine residue. Single-crystal X-ray diffraction (SC-XRD) also confirmed the structures of synthetic **1a**–**d**. No biological studies were reported.

(−)-Himastatin (**4**, Figure 2) is a natural, homodimeric cyclic peptide isolated from *Streptomyces himastatinicus*, which is reported to exhibit antibiotic and antitumor activity [31,32]. The mechanism of action for himastatin is still unknown but is speculated to involve the disruption of bacterial membranes. The homodimer is joined by C_sp2_-C_sp2_ aromatic linkage between the C5-C5’ cyclotryptophan moieties; this dimerization has been tested as crucial for Gram-positive antibiotic activity. Regarding the hybrid solid-solution phase synthesis of **4**, the linear precursor **6** was elaborated by standard fluorenylmethoxycarbonyl (Fmoc)-protected SPPS, starting with on-resin d-Thr, mediated by HATU. Macrolactamization was performed with HATU and 1-hydroxy-7-azabenzotriazole (HOAt) in a head-to-tail manner between Val and cyclotryptophan. The C_sp2_-C_sp2_ dimerization was performed as the final step, and the conditions were determined following a biosynthetically-inspired single-electron transfer using a Cu(II) catalyst. Dimerizations of **5** to form product **4** were accomplished in yields up to 41% (~40% avg.). In addition to **4**, the enantiomer (+)-himstatin and meso-himstatin were prepared in a two-fold accord for confirming characterizations and biological evaluation. In vitro antibiotic assessment was performed on six Gram-positive bacterial strains: *Bacillus subtilis*; Methacillin-resistant *Staphylococcus aureus* (MRSA); Methacillin-sensitive *Staphylococcus aureus* (MSSA); Vancomycin-resistant *Enterococcus* (VRE) *faecalis*; Vancomycin-sensitive *Enterococcus* (VSE) *faecalis*; and *Streptomyces himastatinicus*. Synthetic **4** showed similar MICs (1 to 2 μg/mL) compared to natural himastatin, while stereoisomers showed nearly the same activity. Additionally, the fluorescently-active probe was manufactured, enabling visualization of membrane disruption [33].

**Figure 3:**
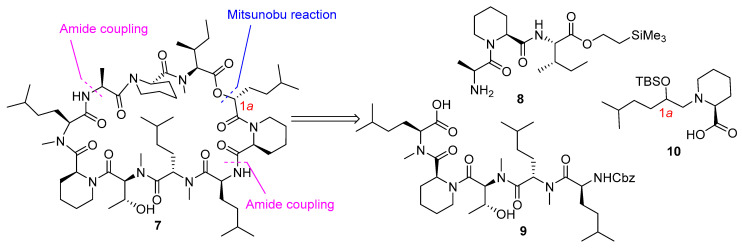
Retrosynthetic analysis of **7**.

The Doi group [34] devised a solution-phase synthesis of natural cyclic peptide decatransin (**7**, Figure 3), a 30-membered cyclic peptide isolated from the saprophyte fungus *Chaetosphaeria tulasneorum* in 2015 [35]. The natural product was reported to have cytotoxic activity against two cancer cell lines (IC_50_ values of 0.14 and 0.03 μM for HCT-116 (human colon) and COS-1 (African green monkey kidney), respectively) based on the uniquely elucidated mechanism of inhibiting co- and post-translocations across the Sec61/SecYEG translocon [36]. Two epimers (position 1α) were synthesized by solution-phase convergent peptide coupling of *N*-alkyl peptide fragments using HATU, EDCI/HOAt, or Bromo-tris-pyrrolidino-phosphonium hexafluorophosphate (PyBrOP). Head-to-tail macrolactonization was accomplished under modified Mitsunobu conditions, diisopropyl azacarboxylate (DIAD)/triphenylphosphine (PPh_3_), resulting in the *S*-epimer in 37% and the *R*-epimer in 44%. Further, the cyclization yield was enhanced to 65% when tri(4-fluorophenyl)-phosphine was used instead of triphenylphosphine. This was likely due to steric hindrance at the carbonyl in the β-branched Me-Ile residue, which is overcome by the leaving ability of the phosphonium oxy intermediate. Synthetic **7** and (***S***)**-7** were characterized by 1D and 2D NMR studies and UPLC/MS. Synthetic **7**, with *R*-configuration at the 1*a* position, matched the natural decatransin’s chemical shifts and retention times. The cytotoxicity of the natural and synthetic products against HCT-116 cell lines was evaluated using MTT assays. Synthetic **7** exhibited potent activity with an IC_50_ value of 0.019 μM, while natural **7** was superior with an IC_50_ value of 0.0030 μM. Synthetic epimer ***S*-7** showed no cytotoxic activity against the same cell line.

**Figure 4:**
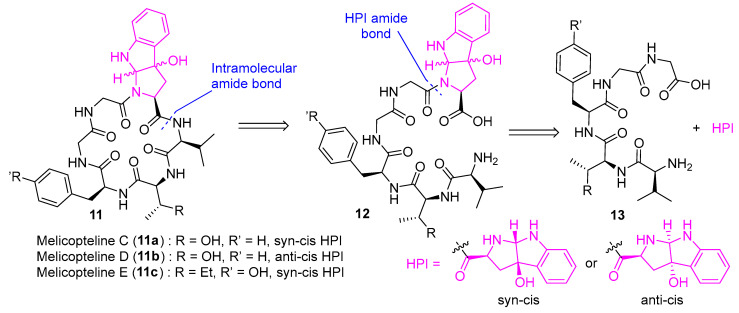
Retrosynthetic analysis of **11a**–**c**.

The plant genus *Melicope* has been used medicinally for centuries in China and Vietnam for symptom relief of colds and the flu [37,38]. New cyclopeptides, called melicoptelines A−E, were isolated from the leaves of *Melicope pteleifolia* by the group of Oh in 2015 [39]. Biological studies revealed melicoptines C-E (**11a-c**, Figure 4), all featuring a hexahydropyrrolo [2,3-b]-indole (HPI) moiety, possess much more potent anti-viral activities compared to melicoptines A and B. Products **11a** and **11c** contain a syn-*cis* HPI, while **11b** features an anti-*cis* HPI moiety. Thus, the two HPI moieties were constructed by cyclizing l-Trp under dimethyldioxirane (DMDO) selevtive oxidation conditions. Convergent solution-phase peptide synthesis was employed by amide couplings using hexafluorophosphate benzotriazole tetramethyl uronium (HBTU) in 7–9 linear steps requiring the synthesis of two different linear peptide intermediates. Each cyclic product was cyclized via head-to-tail macrolactamization between Val and the HPI moiety mediated by HATU/HOAt. The cyclization yields for melicoptines **11a**, **11b**, and **11c** were 67, 65, and 31%, respectively. Antiviral activities were performed on influenza A viruses H1N1 and H9N2. Compound **11a** showed EC_50_ of 4.03 against H1N1 and 12.29 μM against H9N2; compound **11b** showed EC_50_ of 4.60 μM against H1N1 and 8.75 μM against H9N2; compound **11c** showed EC_50_ of 2.88 μM against H1N1 and 7.30 μM against H9N2. These were compared to ribavirin (EC_50_ H1N1 = 2.88 μM; EC_50_ H9N2 = 7.30 μM). A correlation between the viral specificity and differing HPI moieties was observed. Syn-*cis* HPI analogs (**11a** and **11c**) have stronger activities against H1N1, while the opposite is true for anti-*cis* HPI (**11b**), showing stronger, on average, activity towards the H9N2 subtype. Cytotoxicities were evaluated, and all compounds (**11a**–**c**) were not cytotoxic (CC_50_ > 200 μM for all synthesized compounds) [40].

**Figure 5:**
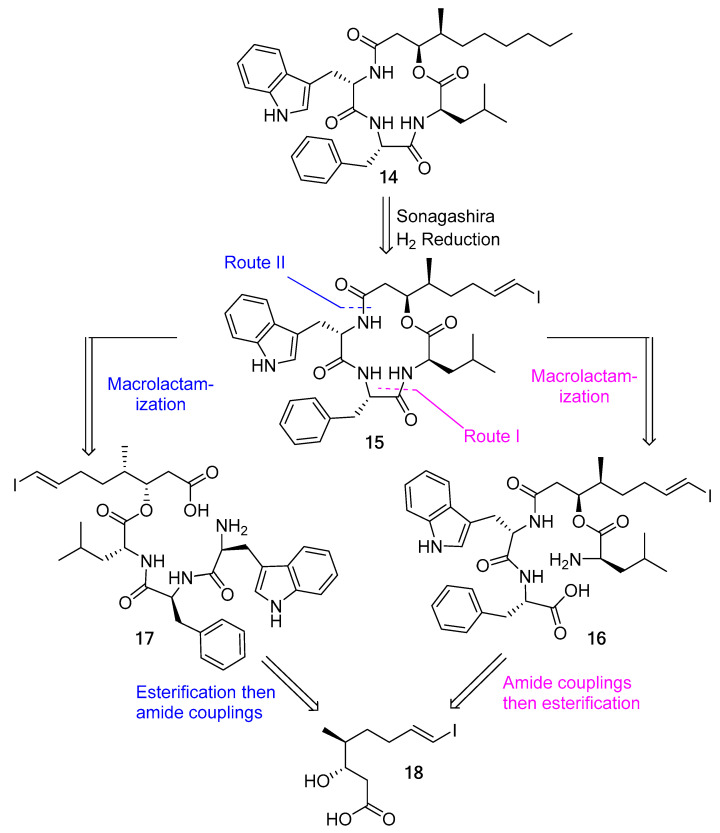
Retrosynthetic analysis of **14**, beauveamide A.

In 2021, Puno and co-workers [41] discovered a family of seven new cyclotetradepsipeptides, beauveamides A−G, when searching for secondary metabolites from fungi using the cultures of endolichenic *Beauveria* sp. isolated from *Gypsoplaca macrophylla* (Zahlbr.) Timdal. Beauveamide A (**14**, Figure 5) was found to be the most biologically active (antitumorigenic) among this family, prompting the Goswami group [42] to attempt its synthesis. Initially, the synthesis relied on a head-to-tail macrolactonization between the Ieu C-terminal and the secondary alcohol at the 3-hydroxy-4-methyldecanoic (HMDA) moiety under various conditions. However, this route was rendered unsuccessful. Figure 5 shows two different macrolactamization routes envisioned, either between the Leu/Phe residues (Route I) and Gly/Trp (Route II). Both approaches were attempted and successful, but the latter proved more efficient due to the preceding esterification of intermediate **16** in Route I. Amide cyclization conditions were optimized using HATU/HOAt and reported up to 78% yields. Late-stage functionalization to install the acid HMDA moiety was completed through a Sonagashira coupling of a vinyl iodide and TMS-protected ethyne, which was subsequently reduced to its alkyl counterpart, resulting in **14**. Antiproliferative activities were assessed using 3-[4,5-dimethylthiazol-2-yl]-2,5 diphenyl tetrazolium bromide (MTT) assays against HeLa (cervical cancer) and MDA-MB-231 (metastatic breast adenocarcinoma) cell lines. Synthetic **14** showed IC_50_ values of 13.6 and 16.2 μM against HeLa and MDA-MB-231, respectively. These were compared to the positive control doxorubicin (HeLa IC_50_ = 5.5 μM and MDA-MB-231 IC_50_ = 8.0 μM).

**Figure 6:**
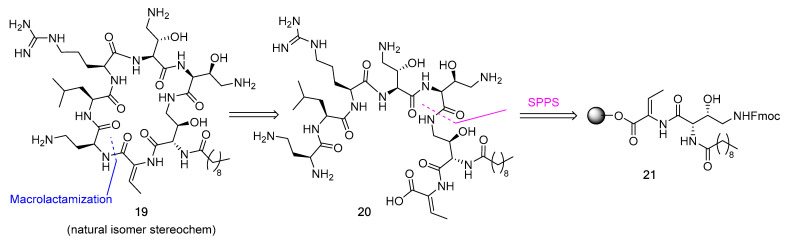
Retrosynthetic analysis of natural product **19**, ogipeptin A.

Kozuma et al. isolated and identified ogipeptins A−D from the marine bacterium *Pseudoalteromonas* sp. SANK 71903 in 2017 [43]. The isolates were tested in vitro as lipopolysaccharide (LPS) inhibitors and potential as Gram-positive antimicrobial agents [44]. Ogipeptins consist of three consecutive β-hydroxy-α,γ-diaminobutyric acids (β-OH Dabs) residues. Still, SC-XRD analysis, among other methods, could not determine their absolute configurations. The Nishi group [45] sought to determine the stereochemical identity of the active compound in ogipeptin A (**19**, Figure 6) extracts by generating an efficient synthesis of all three possible diasteriomers at the β-OH Dabs residues. Following the synthesis and resin loading of chiral building blocks (**21**), the linear precursors **20** were separately achieved through standard Fmoc-SPPS protocol using a chlorotrityl chloride resin. Linear peptides **20** were elaborated in the presence of HATU, resulting in ~30% yields after high-performance liquid chromatography (HPLC) purification. Head-to-tail cyclization was completed in the solution phase using HATU under diluted conditions (1 mM, acetonitrile (ACN)/tetrahydrofuran (THF)) between 2,4-diaminobutyric acid tail and 2-amino-2-butenoic acid head. The three diastereomers were isolated in yields between 16–19% (cyclization + deprotection). HPLC retention times and ^1^H/^13^C nuclear magnetic resonance (NMR) data of **19** were compared to the natural product and were in good agreement. No biological studies were performed on the compounds.

**Figure 7:**
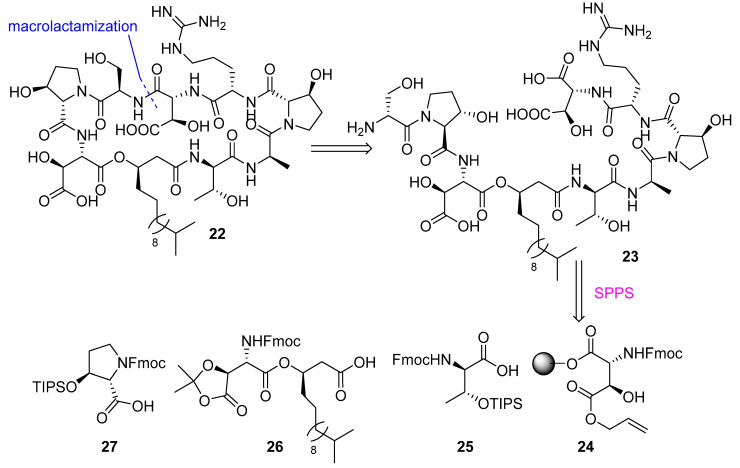
Retrosynthetic analysis of **22**, plusbacin A_3_.

Lipodepsipeptide natural products, such as plusbacin A_3_ (**22**, Figure 7), exhibit antibacterial activity by inhibiting bacterial cell wall biosynthesis in a wide range of Gram-positive bacteria and is presumed not to cause bacterial resistance [46,47]. Plusbacin A_3_ was first isolated from a *Pseudomonas* species, which is characterized by nonproteinogenic amino acids, including *trans*-3-hydroxy-l-Pro (3-HyPro) and β-threo-hydroxy-Asp (HyAsp) [48]. The total synthesis of **22** was previously reported as a solution-phase synthesis [49], but Takashina et al. [50] sought to achieve the synthesis using the solid-phase protocol, enabling future derivatization of **22**. First, a diastereodivergent Joullie−Ugi three-component reaction (JU-3CR) was utilized in the first step in synthesizing the necessary building blocks **24**–**27**. Using these building blocks, the linear peptide was synthesized using chlorotrityl resin in the presence of HATU. Cyclization was carried out using Benzotriazole-1-yl-oxy-tris-pyrrolidino-phosphonium hexafluorophosphate (PyBOP) and HOAt in solution, affording the head-to-tail cyclized product in 30% yield, which was subsequently deprotected to give **22**. In vitro antibacterial studies were performed on two strains of *S. aureus* (ATCC 25923 and JE2 MRSA) with minimum inhibitory concentrations (MICs) of 1 μg/mL for both strains and three strains of *E. faecium* (ATCC 35667, VRE ATCC 51299, and VRE ATCC 51559) showing MICs of 8, 2, and 16 μg/mL, respectively. Notably, **22** showed good activity against MRSA and remained sensitive to VRE [51].

**Figure 8:**
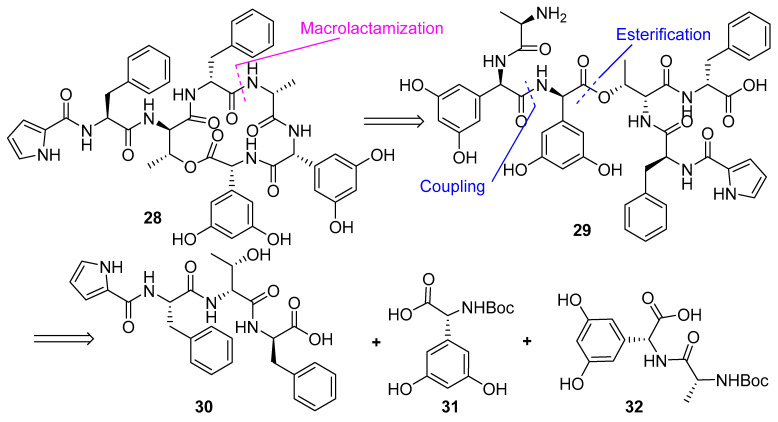
Retrosynthetic analysis of **25**, cochinmicin I.

The cyclodepsipeptides cochinmicin I−V isolated from *Microbispora* sp. ATCC 55140 has been reported as an endothelin receptor antagonist (ERA), which could influence blood pressure [52]. Endothelin receptor subtypes A (ET_A_) and B (ET_B_) are involved in vasoconstriction and vasodilation, respectively, and previous reports on cochinmicins showed notable IC_50_ values in vitro [53]. Cochinmicin I (**28**, Figure 8) was the most potent among the isolated cochinmicins and can potentially act as a cardiovascular agent for heart disease [54]. The Sussmuth group [55] devised a solution-phase total synthesis, starting with developing a pentapeptide precursor from intermediates **30**–**32**. Once functionalized, pentapeptide **29** was subjected to head-to-tail macrolactamization using 3-(diethoxyphosphoryloxy)-1,2,3-benzotriazin-4(3H)-one (DEPBT) and NaHCO_3_ resulting in **28** in 45% yield after deprotection. The product was characterized by NMR (^1^H/^13^C) and HPLC methods to establish standards and compare to its naturally sourced counterpart. The spectra of **28** were in good agreement with natural cochinmicin I. Synthetic **28** was then tested for antiviral and antifungal activity and their effect on voltage-gated Na^+^/K^+^ channels, but their results were not noteworthy and, therefore, not reported.

**Figure 9:**
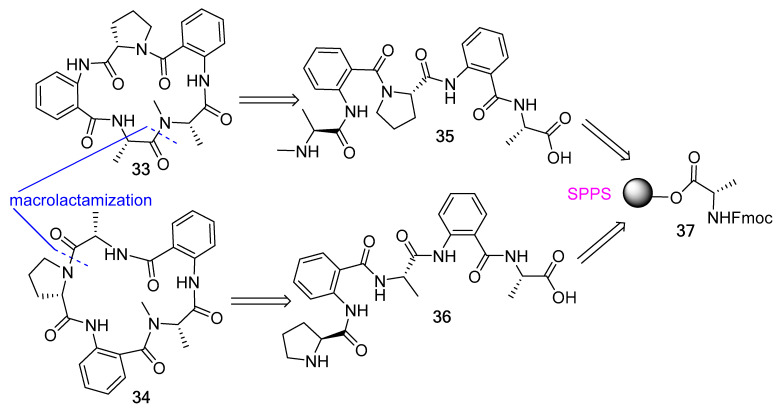
Retrosynthetic analysis of compounds **33** and **34**, versicotides E and F, respectively. Macrolactamization was performed in a head-to-tail directionality from linear precursors **35** and **36**. These linear peptides were synthesized through SPPS from the common intermediate **37**.

The Serra group reported the total synthesis of versicotides E and F (**33** and **34**, Figure 9) via head-to-tail cyclization [56]. Versicotides E and F have been isolated from *Aspergillus versicolor* LZD-14-1 [57], featuring an anthranilic acid moiety, and have been identified as potential phytotoxins [58] and inhibitors of cyanobacteria [59]. SPPS with Fmoc protection was utilized to achieve both linear analogs, **35** and **36**, from the common loading intermediate **37**, using *N,N′*-Diisopropylcarbodiimide (DIC) and Ethyl cyano(hydroxyimino)acetate (Oxyma). Solution-phase cyclization was completed in 2 days, mediated by HATU, leading to **33** and **34** in 20% and 17% yield, respectively. Phytotoxicity studies were performed on ryegrass species Lolium multiflorum, revealing that both products inhibited ryegrass growth but did not prevent germination. Additionally, vericotides A-F were tested against colonies of the cyanobacteria species *Microcystis aeruginosa*, showing potential as cyanobacteria growth inhibitors.

**Figure 10:**
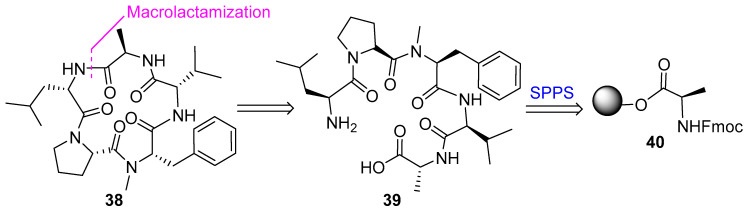
Retrosynthetic analysis of **38**, xylapeptide B.

Xylapeptide B (**38**, Figure 10) is a cyclopentapeptide, first isolated from *Xylaria* sp. derived from the Chinese medicinal plant *Sophora tonkinensis* [60]. It was reported to have high antibacterial activity against *Bacillus subtilis* and *Bacillus cereus* with respective MIC values of 12.5 and 6.25 μg/mL. Xylapeptide B also showed antifungal activity against *Candida albicans* with an MIC value of 12.5 μg/mL. Kurnia et al. [61] devised a total synthesis of **38** using both solid and solution phase synthesis. Solid-phase chlorotrityl chloride resin was used to synthesize the linear intermediate **39**, mediated by HBTU/HOBt, resulting in an 18.8% yield of **39**. Macrolactamization involved coupling the l-Leu with d-Ala using HBTU/HOBt in 1% DIPEA (%*v*:*v*) at 1.25 mM in dichloromethane (DCM). Semipreparative RP-HPLC was used for purification, giving product **38** an 8.9% yield. The product was characterized and confirmed by ^13^C NMR and ^1^H NMR, high-resolution time-of-flight mass spectroscopy (HR-ToFMS), and analytical reverse-phase (RP)-HPLC. No biological studies were conducted on the synthetic product.

**Figure 11:**
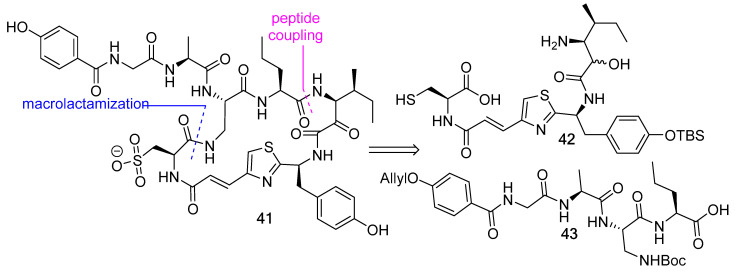
Retrosynthetic analysis of **41**, cyclotheonellazole A.

Acute lung injury/acute respiratory distress syndrome (ALI/ARDS) is mediated by elastase, a Ser protease expressed in neutrophils [62]. Therefore, elastase inhibitors could be a potential target for severe pulmonary infections/diseases [63]. Cyclotheonellazole A (**41**, Figure 11) was first isolated from the marine sponge *Theonella affinis swinhoei* by Carmeli et al. [64] in 2017 and was reported to significantly inhibit elastase (IC_50_ value of 0.034 nM) in vitro. The synthesis developed by Cui et al. [65] utilized only solution-phase convergent synthesis by synthesizing two tetrapeptide fragments, **42** and **43**, to afford the linear precursor. Cyclization was carried out in a head-to-tail orientation using HOBt/EDCI, reporting a 60% yield (Gram-scale). After several post-macrocyclic modifications, **41** was achieved. Characterization of **41** with ^13^C/^1^H NMR and mass spectrometry matched the reported characterizations of natural Cyclotheonellazole A. In vitro biological studies were performed on porcine pancreatic elastase (PPE) and human neutrophil elastase (HNE), and **41** showed IC_50_ values of 0.114 and 0.321 μM, respectively. Notably, in vitro studies of **41** outperformed sivelestat (positive control) on the same cell lines (IC_50_ values of 0.704 and 2.96 μM, respectively). Additionally, in vivo mouse studies were conducted. They showed improved acute lung injury on bleomycin-induced mice when **41** was administered at 30 mg/kg/day, while in vitro cytotoxicity assays showed no cytotoxicity up to 100 μM concentrations.

**Figure 12:**
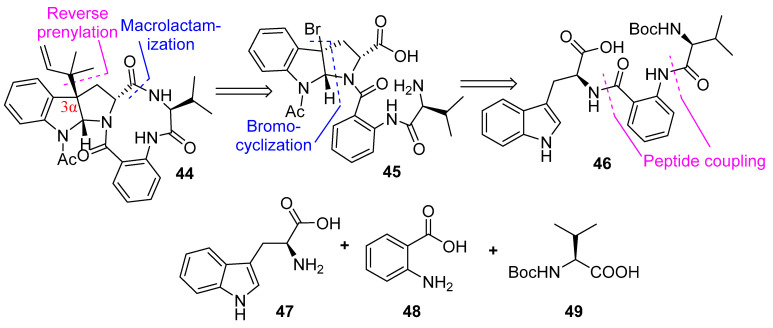
Retrosynthetic analysis of **44**, (−)-Novofumigatamide.

The Lera group [66] strategized the synthesis of (−)-novofumigatamide (**44**, Figure 12), a fungal secondary alkaloid metabolite presumed to possess biological activity. In 2010, Hosoe et al. [67] extracted **44** from the CBS117520 strain of the fungus *Aspergillus novofumigatus*. The analyzed sample showed the presence of a hexahydropyrrolo[2,3-*b*]indole skeleton bearing a reverse-prenyl group at the C3α position. Although no significant antimicrobial or cytotoxic activity was initially found for **44** [67], further evaluations could be plausible following a reliable synthesis. Initial synthetic intervention accomplished the proposed structure, but the NMR characterization did not match the natural counterpart. New synthetic routes were subsequently implemented, each differing in the sequence of the final three steps. The successful synthesis shown in Figure 12 (showing the proposed stereochemistry of **44**) of all possible enantiomers and corresponding diastereomers occurred through convergent amide couplings of starting materials **47**–**49**. Subsequently, a diastereoselective bromocyclization was utilized to form the hexahydropyrrolo[2,3-*b*]indole moiety. Following deprotections, HATU-mediated head-to-tail macrolactamization was accomplished at 3 mM, resulting in up to 97% yields. A reverse prenylation at the C3α position resulted in **44**. Although NMRs and specific rotations of two synthesized products obtained were identical to the natural product, the authors believe further intense structural intervention is needed to elucidate the correct connectivity of natural **44** fully.

**Figure 13:**
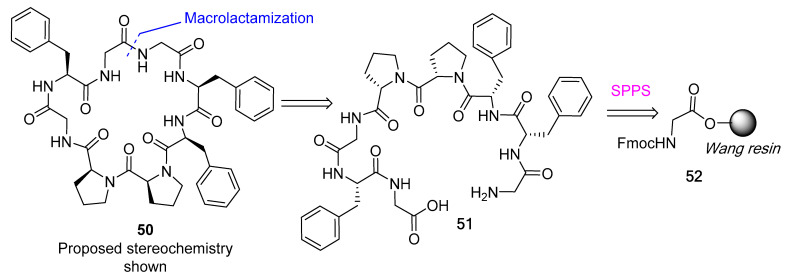
Retrosynthetic analysis of **50**, [1-8-NαC]-zanriorb A1.

Orbitides are described as ribosomally synthesized and post-translationally modified peptides (RiPPs), linked from head-to-tail and lacking disulfide bridge(s) found in many plant species [68]. Natural [1-8-NαC]-zanriorb A1 (**50**, Figure 13) is an orbitide found in the plant *Zanthoxylum riedelianum*. It has been used in Brazilian medicine as an analgesic. It has been shown to induce apoptosis in Jurkat leukemia T cells and to be highly cytotoxic with an IC_50_ of 218 nM [69]. The Shaheen group [70] attempted to elucidate a procedure for both solution-phase and solid-phase cyclizations of **50** and compare synthetic efficacy and biological results to the natural counterpart. The linear peptide precursor was achieved via solid-phase synthesis using a Wang resin and Fmoc-protected amino acids. SPPS resulted in the crude linear peptide **51** in 55% yield mediated by PyBOP. RP-HPLC purified the linear peptide. A solution-phase cyclization of 51 was carried out using HATU/DIPEA, which gave a 22% cyclization yield. An attempted solid-phase cyclization utilized a ‘safety-catch’ resin, 4-Sulfamyl butyryl-aminomethyl, which is stable while assembling the linear peptide but is labile under alkylating conditions promoting intramolecular cyclization of the free amine tail. The linear peptide was constructed under the same coupling conditions but loaded onto the aforementioned resin. The sulfonamide was alkylated with ICH_2_CN/DIPEA/*N*-methyl-2-pyrrolidone (NMP). However, the cyclized products from the on-resin macrocyclization showed low yields of two rotamers. The fast atom bombardment mass spectroscopy (FABMS) and NMR studies of the cyclized product from solution-phase cyclization matched that of the reported natural counterpart. However, it was determined that there was a mixture of rotamers within their synthetic product and the natural product. Based on Distortionless Enhancement by Polarization Transfer (DEPT) 135 ^13^C-NMR spectra, the major rotamer was determined to be *trans*, *cis*-rotamer the Pro^1^-Pro^2^ and Pro^2^-Phe^3^ amide bonds, respectively. Synthetic **50** did not show cytotoxicity previously observed. The authors reasoned this could be secondary to an impure isolate.

**Figure 14:**
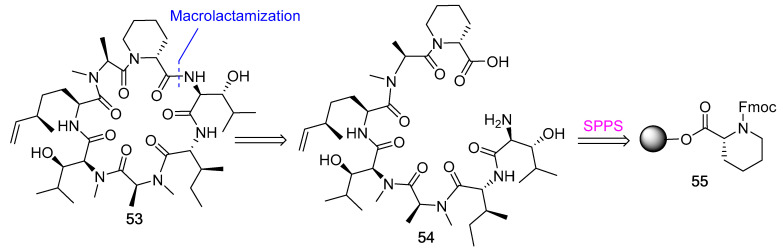
Retrosynthetic analysis of **53**, A3.

Heptapeptide A3 (**53**, Figure 14) was isolated from an *Aspergillus* fungal strain in 2010 by Blunt et al. and established its ability to inhibit the proliferation of human cancer cells at low nanomolar concentrations [71]. The natural peptide belongs to the ternatin family of cyclic peptides, featuring dehydromethyl Leu (dhML) and pipecolic acid residues. Although the amino acid sequence was elucidated, the absolute configuration of several residues was not assigned. Thus, Wang et al. [72] established a total synthesis of **53** to determine the configurations at the unknown stereogenic centers. To begin, the dhML building block **55** was first synthesized through a series of three steps and used in SPPS. HATU-mediated SPPS resulted in linear precursor **54**. After global deprotection, cleavage, and purification, head-to-tail cyclization of the linear peptide was carried out in the solution phase, also using HATU under pseudo-diluted conditions (two syringe pumps, 1.8 mL/h). The overall yield was calculated at 56%. Two epimers resulted at the dhML moiety (*S,S*-dhML and *S*,*R*-dhML), which were characterized and compared to natural A3. The absolute configuration of diastereomer **53** containing (*S*,*R*)-dhML matched the natural product by both NMR and HPLC retention time. Preclinical trials revealed intermittent, low doses of **53** (1.5–2.0 mg/kg, three times per week), profoundly reduced tumors, and extended the survival of Eμ-Myc mice without toxicity.

**Figure 15:**
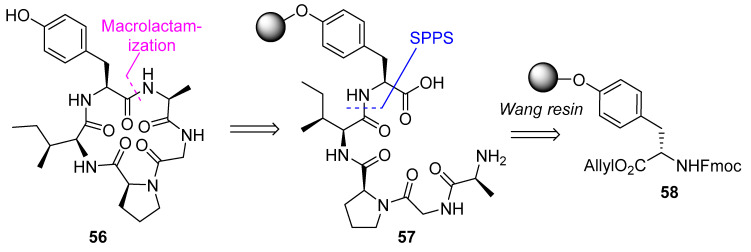
Retrosynthetic analysis of **56**, fanlizhicyclopeptide B.

A total synthesis of fanlizhicyclopeptide B (**56**), an implicated anti-inflammatory agent, was completed by Cui et al. [73]. Natural **56** was first extracted by Wu et al. in 2014 from the fruit (sugar apple) of *Annona squamosa* L. and gained attention for its analgesic properties [74]. Subsequent studies identified **56** as inhibiting TNF-α and IL-6, further implicating its possible medicinal application(s). The synthesis of **56** (Figure 15) was initiated using a Wang resin for a common Fmoc-protected SPPS protocol. An allyl-protected Tyr (**58**) was loaded onto the Wang resin using Mitsunobu conditions, and the linear peptide was elongated with the aid of 2-(6-chloro-1-H-benzotriazole-1-yl)-1,1,3,3-tetramethylaminium hexafluorophosphate (HCTU) and in the presence of Hunig’s base. The on-resin linear peptide **57** was subsequently deprotected and cyclized head-to-tail using 7-azabenzotriazol-1-yloxy)tripyrrolidinophosphonium hexafluorophosphate (PyAOP) and HOAt in the presence of *N*-methylmorpholine (NMM). Following resin cleavage and RP-HPLC, **56** was obtained in reported yields of 57% and characterized using high-resolution electron spray ionization mass spectroscopy (HR-ESI-MS) and NMR. No biological studies were reported.

**Figure 16:**
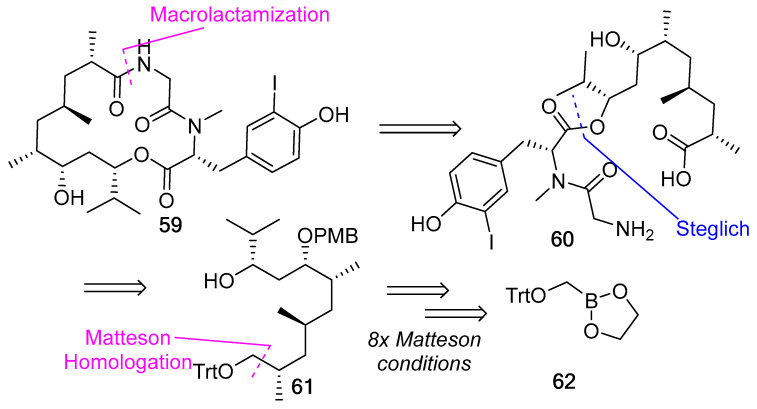
Retrosynthetic analysis of **59**, doliculide.

Doliculide (**59**, Figure 16) is a natural depsipeptide isolated from the sea hare *Dolabella Auricularia* [75] that has been implicated as a potential anticancer agent [76]. Natural **59** features a polyketide moiety, also seen in the structurally related cyclic peptide jasplakinolide, which has shown substantial antiproliferative activity against several cancer cell lines [77]. The Kazmaier group [78] devised a total synthesis of **59** utilizing a Matteson homologation in the initial stages (**62**) to generate the polyketide fragment **61**. This unit underwent Steglich esterification with a modified Gly-Tyr dipeptide using EDCI/DMAP/Collidine in good yield to achieve linear precursor **60**. Cyclization conditions were optimized for head-to-tail lactamization between the polyketide head/Gly using benzotriazole-1-yl-oxy-tris-(dimethylamino)-phosphoniumhexafluorophosphate BOP at a 0.25 mM concentration, resulting in a 77% cyclization yield. Cyclization between Gly and Tyr residues was also investigated to mitigate epimerization. This was accomplished using Bis(2-oxo-3-oxazolidinyl)phosphinic chloride (BOPCl) at the diluted concentration (vide supra), giving a 75% yield. In addition to **59**, four analogs were synthesized for structure-activity relationship (SAR) studies. Cytotoxicity studies were carried out against five cancer cell lines: HCT-116, U-2 OS (human bone), Hep2G (human liver), KB3.1 (human cervix), and CHO-K1 (Chinese hamster ovary). Observed IC_50_ values for synthetic **59** were reported in the nanomolar range: 8.8, 25.1, 16.4, 38.0, and 110.8 nM, respectively.

**Figure 17:**
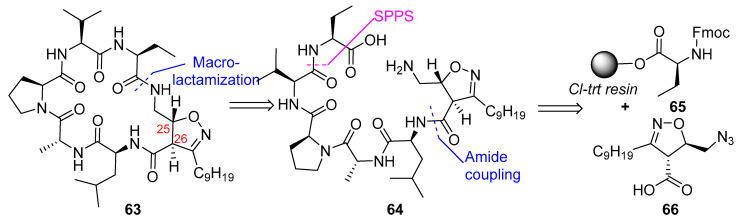
Retrosynthetic analysis of **63**, mutanobactin D. C25 and C26 (red) were unassigned stereocenters prior to the report.

Mutanobactin D (**63**, synthetic analog) is a non-ribosomal cyclic peptide isolated from *Streptococcus mutans* that has been shown to reduce yeast-to-hyphae transition as well as biofilm formation of the opportunistic pathogenic yeast, *C. albicans* [79]. Mutanobactins could, therefore, serve as biofilm-preventing agents in the realm of surgical and medical devices, reducing the excessive use of common antifungal agents, which are usually inactive against biofilms. Pultar et al. [80] established the first total synthesis of **63** (Figure 17), which contains a β-hydroxy-β′-keto-γ-amino amide subunit part of the lipopeptide domain. Additionally, the absolute configurations at C-25 and C-26 were inconclusively reported in the past [81] and remained unknown. In the solution phase, the azido carboxylic acid moiety was synthesized with a dipolar cycloaddition reaction of ethyl 4-hydroxycrotonate and the nitrile oxide generated in situ from hydroximoyl chloride. The linear peptide precursor was elongated using Fmoc-SPPS with a chlorotrityl resin using HATU. However, attachment of the azido carboxylic acid residue required DIC/HOBt in base-free, solution-phase conditions, resulting in the linear hexapeptide **64** in 85% yield. Cyclization was accomplished via head-to-tail lactamization, following a Staudinger azide reduction, giving a free primary amine (PMe_3_ THF *aq.*), using PyAOP/HOAt. It resulted in a 75% cyclization yield after HPLC. Reductive hydrolysis resulted in the proposed structure of **63**, which matched the reported NMR data of the naturally sourced product. The stereochemical assignment was carried out by stereocontrolled synthesis in combination with derivatization studies, J-coupling analysis, and isotopic labeling experiments. These were further confirmed by applying an infrared sequence alignment (IRSA) algorithm in combination with quantum chemical calculations. Biofilm formation assays (XXT) were completed with **63** against three different strains of *C. albicans* (ATCC 90028, 101, and SC5314) with IC_50_ values recorded following reported assays (IC_50_ = 23.5, 24.7, and 21.5 μM, respectively).

**Figure 18:**
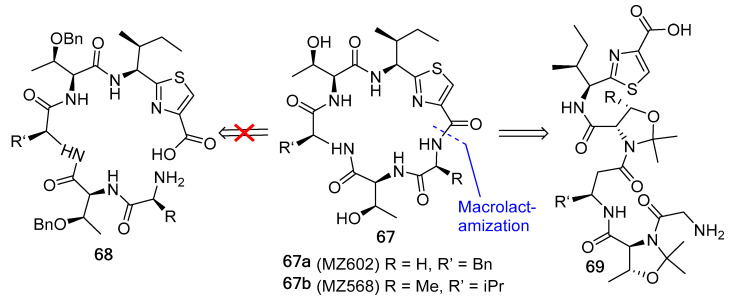
Retrosynthetic analysis of **67** macrolactamization step. Linear precursor **68** failed to cyclize with benzylic protecting groups on each Thr residue. Cyclization was only successful with pseudoproline (Ψ^Me,Me^ Pro) protecting groups seen in **69**.

Microcyclamides MZ602 and MZ568 (**67a**,**b**, Figure 18) are two cyclic hexapeptide secondary metabolites of cyanobacteria isolated from the freshwater *Microcystis* sp. in 2010 by Carmeli and co-workers [82]. The structure was elucidated by ^1^H NMR, ^13^C NMR, and UV methods, while Marfrey’s method determined the absolute configurations to be all l-amino acids. In Figure 18, MZ602 (**67a**) and MZ568 (**67b**) differ at positions C-1 and C-5, where **67a** has a Gly and Phe at C-1 and C-5, respectively, and **67b** has Ala and Val residues, respectively. Preliminary biological screening showed that microcyclamide MZ602 (**67a**) exhibited mild inhibitory activity against the Molt4 T-cell line (20% cell growth inhibition at 83 μM) and moderate inhibitory activity against chymotrypsin (IC_50_ = 75 μM). Compared to MZ602, microcyclamide MZ568 (**67b**) showed a more potent inhibitory activity against Molt4 cells with cell growth inhibition of 36% at 1.8 μM, but not active against chymotrypsin [82]. The Du group [83] devised a total synthesis of each cyclic peptide, each containing a single thiazole ring within the structure. Assembling each linear precursor was achieved using a three-component solution-phase convergent synthesis method. The first attempt of cyclizing linear precursors **68a**,**b** utilized benzylic protecting groups on each Thr residue. Under several coupling conditions, products **67a**,**b** were not observed. Macrolactamization was only successful using linear analog **69**, containing pseudoproline (Ψ^Me,Me^ Pro) protecting groups. The authors reasoned that this enhanced *cis*-amide bond formation and promoted cyclization in a head-to-tail manner. Optimized conditions for cyclization used HATU/HOBt, reporting overall yields (over 11 linear steps) for **67a** and **67b** were 12.5% and 16.8%, respectively. Spectral data (^1^H NMR, ^13^C NMR, and HRMS) of **67a** showed good agreement with the naturally sourced product data but differed in the specific rotation. Spectral data and specific rotations of **67b** showed very different characterizations. However, SC-XRD of the linear precursor to **67b** confirmed all l-amino acids and connectivity. The authors conclude that revisiting the proposed structures of the naturally sourced compounds MZ602 and MZ568 is needed.

**Figure 19:**
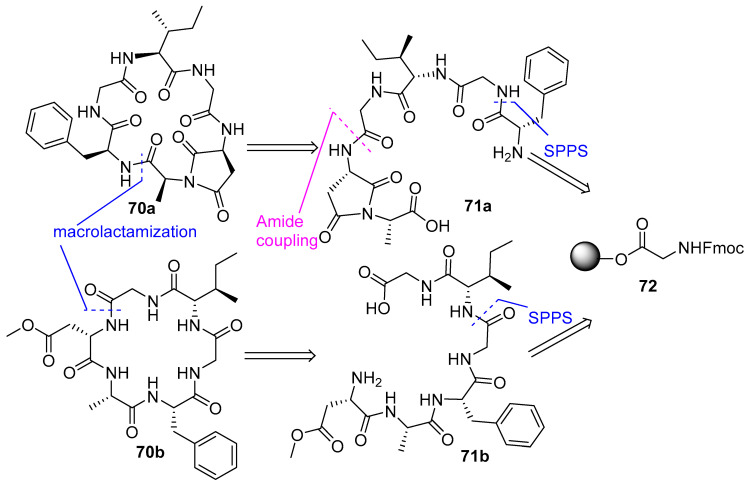
Retrosynthetic analysis of macrocyclization of products **70a** and **70b**. Each linear precursor, **71a** and **71b**, elaborated using SPPS from a common on-resin Gly analog **72**. Macrolactamization of each linear analog resulted in each respective cyclized product, **70a** and **70b**.

Nitidumpeptins A and B (**70a** and **70b**, Figure 19) are natural cyclic hexapeptides isolated from the herb *Zanthoxylum nitidum* var. *tomentosum*. The Liang group conducted the isolation, characterization, and synthesis of both products [84]. Structural elucidation was established by both 1D and 2D NMR (COSY, HSQC, HMBC) along with HR-ESI-MS. Each 18-membered cyclopeptide contains six amino acid residues; **70a** features a unique pyrrolidine-2,5-dione unit within its structure. Marfey’s method and ESI-MS/MS presumed the absolute configurations, finding all amino acid residues in both structures to be in the l-configuration. Total syntheses for each were described to confirm their absolute configurations and devise a scalable production. Linear precursors **71a** and **71b** were prepared separately using standard Fmoc-protected SPPS, mediated by 2-(1*H*-benzotriazole-1-yl)-1,1,3,3-tetramethylaminium tetrafluoroborate (TBTU), starting with the common on-resin intermediate **72**. Cleavage and deprotection resulted in each linear peptide (**71a** and **71b**), which were purified by preparative HLPC methods to separate a resulting epimer of each. Cyclization of both linear precursors took place in the solution phase using head-to-tail macrolactamization with HATU under highly diluted conditions: 0.5 mM. Cyclization yields were reported at 65% (**70a** and **70b**), and both products were characterized by NMR and structurally confirmed to their naturally sourced counterparts. Antiproliferative activities of **70a** and **70b** were evaluated against human gefitinib-resistant non-small cell lung cancer (NSCLC) cells (HCC827-gef cells). The results were insignificant, showing little cytotoxicity from each cyclic peptide. However, when combined with gefitinib, **70b** showed synergistic activity against HCC827-gef cells, exhibiting enhanced cytotoxicity. It was suggested that **70b** could be an inhibitor of yes-associated protein (YAP), a protein upregulated in certain cancer cells. In vitro assays and docking studies found evidence to support this. Biological assays found the IC_50_ values of **70b** (+ gefitinib) were 0.98 μM, while gefitinib alone showed an IC_50_ of 6.39 μM. The results suggested that **70b** could be a synergistic sensitizer for some chemotherapies.

**Figure 20:**
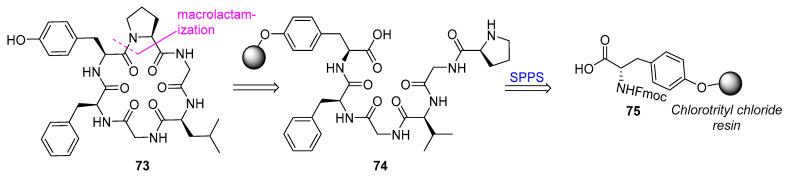
Retrosynthetic analysis of natural cyclic peptide cherimolacyclopeptide E, **73**. The linear precursor **74** was elaborated using SPPS, starting with resin-loaded intermediate **75**. Macrolactamization of **74** was elected between Tyr and Pro head-to-tail, respectively, giving **73**.

Cherimolacylopeptide E (**73**) is a cyclic hexapeptide isolated from the seeds of *Annona cherimola* by Wélé et al. [85,86]. Reportedly, naturally sourced **73** exhibited potent nanomolar cytotoxicity against KB human nasopharyngeal carcinoma cells (IC_50_ 0.017 μM) [87,88,89]. Although previous reports [90,91] have attempted a total synthesis for **73**, aspects of their final characterizations were inconsistent or inconclusive compared to natural **73**. Therefore, Yoshida et al. [92] devised a reliable synthesis of **73** with a reported overall yield of 16%. As seen in Figure 20, the synthesis was carried out entirely on a chlorotrityl chloride resin, with SPPS elongation mediated by DIC/HOBt starting with Tyr intermediate **75**. The resin-bound linear peptide **74** was subject to head-to-tail macrolactamization at the C-terminal Tyr and N-terminal Pro residues in the presence of HATU. Following resin cleavage, synthetic product **73** was characterized by ^1^H/^13^C NMR, ESI-MS, and subject to Marfrey’s method (further confirming the absence of epimers). The characterizations revealed the presence of two conformers, the major of which was in good agreement with the reported spectra of natural **73**. The same biological evaluations were performed as in previous reports [87,89,91]. Still, the results showed little cytotoxicity for **73** against KB cells (IC_50_ > 100 μM) and essentially no activity against *E. coli* (MIC > 10,000 μg/mL). Therefore, the biological application of such a class of cyclic peptides is to be elucidated.

**Figure 21:**
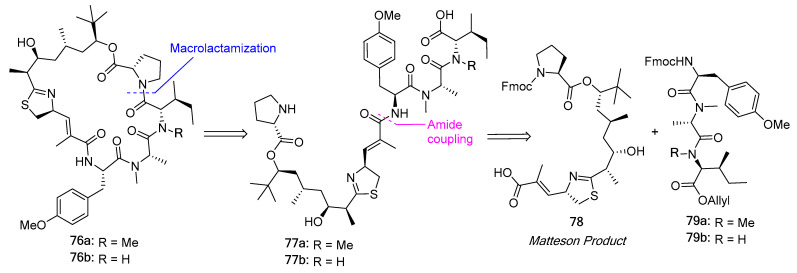
Retrosynthetic analysis of macrocyclization of natural cyclic peptide apratoxins A and B, **76a** and **76b**. The Matteson product **78** was coupled with each respective tripeptide **79** to give **77a** and **77b**. Macrolactamization of precursor **77** was elected between Ile head and Pro tail, resulting in **76a**,**b**.

The apratoxins A–D are a class of cyclodepsipeptides produced by cyanobacteria [93,94,95,96], first isolated from *Lyngbya majuscula* in 2001 was apatoxin A by Moore and Paul et al. [97]. Biological in vitro testing showed potent cytotoxicity toward a range of tumor cell lines in the sub-nanomolar range [98,99]. Several apratoxins have also been isolated (e.g., apratoxins B-D, S4, S10) [100] but are less studied. Andler and Kazmaier [101] devised a total synthesis of both apatoxins A (**76a**) and B (**76b**). The two products differ only in an *N*-methylation at the Ile residue where **76a** is methylated, and **76b** is not. The synthesis was carried out exclusively in the solution phase, initiated by a Matteson homologation to asymmetrically elongate the polyketide carbon chain into a usable precursor **78** (Figure 21). Two tripeptides (**79a** and **79b)** were individually coupled to **78**, resulting in a linear **77a** and **77b** precursors. Head-to-tail macrolactamization was completed between an N-terminal Pro residue and the [*N*-methyl]-Ile carboxylic acid C-terminal using HATU, resulting in **76a** and **76b** in 34 and 36% yields. Preparative HPLC was used to purify each product, and characterizations matched both natural **76**. No biological studies were reported.

**Figure 22:**
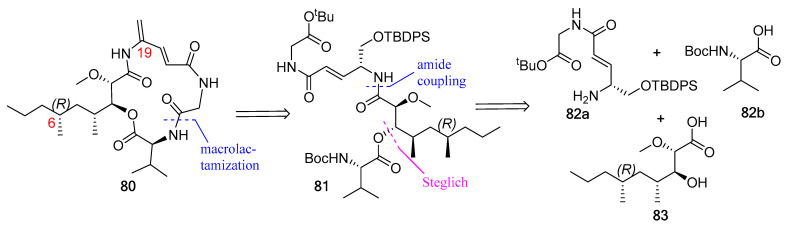
Retrosynthetic analysis of **80**.

Boholamide A (**80**, Figure 22) was first isolated in low yields from the *Mollusca* sp. *Truncatella* by Torres et al. in 2020 [102]. Preliminary biological screening showed nanomolar cytotoxicities (100 to 400 nM) against cancer stem cells [102]. Han et al. [103] sought to produce a convenient total synthesis of **80** to confirm its structure and further explore its therapeutic potential. Natural **80** is a cyclotetradepsipeptide connected by a 4-amino-2,4-pentadienolate (APD) moiety. A convergent solution-phase synthesis employed three separate fragments (**82a**, **82b**, and **83**). Intermediates **82a** and **82b** were coupled to **83** by way of an amide coupling and Steglich esterification, respectively. Macrolactamization of linear precursor **81** was carried out in a head-to-tail orientation mediated by HATU at 1 mM, resulting in yields of up to 91% (Gram-scale). Synthetic **80** was isolated following an elimination reaction at C19. The structural intervention was performed after the post-cyclization modifications were completed, which showed structural inconsistencies between the reported boholamide A and synthetic **80**. Further evaluation revealed that the absolute configuration at C6 was incorrectly reported as (*S*)-CH_3_. The revised structure of boholamide A at C6 was achieved using the same synthetic conditions giving (*R*)-CH_3_ (C6, Figure 22). Natural boholamide A showed superior cytotoxic activities compared to synthetic **80**. However, synthetic **80** showed enhanced cytotoxic activity under hypoxic conditions: 1.92 μM (normoxia) and 0.96 μM (hypoxia).

**Figure 23:**
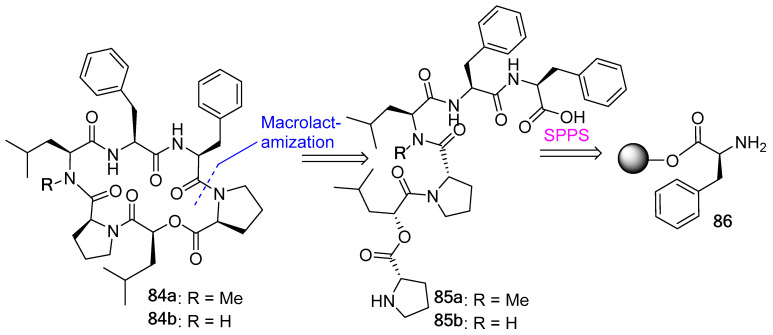
Retrosynthetic analysis of natural cyclic peptide exumolides A and B, **84a** and **84b**. Common intermediate **86** initiated SPPS for each peptide. Linear intermediates **85a** and **85b** were cyclized head-to-tail between Phe and Pro residues.

Exumolides A and B (**84a** and **84b**, Figure 23) are cyclic hexadepsipeptides, previously isolated from the marine fungus of genus *Scytalidium*, shown to inhibit chlorophyte (*Dunaliela* sp.) growth [104]. Structurally, each product contains the same sequence of l-amino acids, while **84a** is an *N*-methylated version of **84b** at the Leu residue. Both compounds contain a single hydroxy acid moiety, (*S*)-2-hydroxy-4-methylpentanoic acid (Hmp). Maharani and co-workers [105] reported a hybrid solid- and solution-phase total synthesis of each natural product, using SPPS and solution-phase cyclization. The Hmp moiety was constructed first through a diazotization reaction between l-Leu and sodium nitrite under acidic conditions, resulting in (*S*)-hydroxy-4-methylpentanoic acid. Using a chlorotrityl chloride resin, precursor elaboration was accomplished either in the presence of HBTU/HOBt or HATU/HOAt, starting with on-resin intermediate **86**. The single ester linkage found in each peptide was executed under modified Steglich conditions (DIC/DMAP, on-resin). Solution-phase macrolactamization was completed under basic conditions in the presence of HATU, between the N-terminal Pro and C-terminal Phe, at 10 mM (yield not reported). The purification of each cyclic product was completed using standard phase column chromatography. Subsequent 1H/13C NMR and HR-QTOF-MS characterization agreed with natural **84a**,**b**; RP-HPLC confirmed their purities. Products **84a** and **84b** were synthesized in 4.12 and 6.39% yields, respectively. No biological studies were reported.

**Figure 24:**
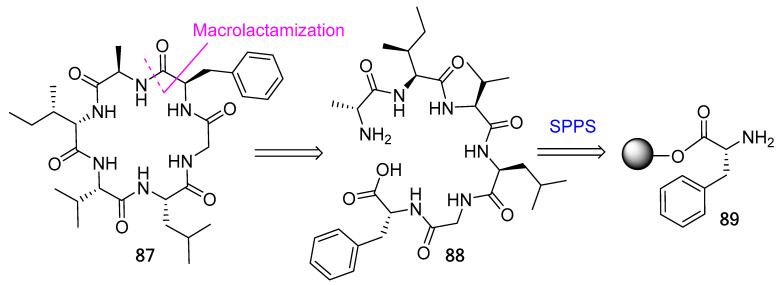
Retrosynthetic analysis of natural cyclic peptide **87**.

The cyclic hexapeptide bacicyclin (**87**, Figure 24) was first isolated from the marine blue mussel *Mytilus edulis* and exhibited antibacterial activity against *E. faecalis* and *S. aureus* (MICs of 8 and 12 μM, respectively) [106]. Chen et al. [107] designed a total synthesis involving solution- and solid-phase techniques with a reported overall yield of 20%. Solid-phase synthesis provided linear precursor **88**, mediated by HCTU on a chlorotrityl chloride resin. Macrolactamization, carried out in the presence of PyBOP/HOBt in DCM in a head-to-tail orientation, was accomplished between Ala and Phe residues (no reported yield). Product **87** was characterized by ^1^H/^13^C NMR and HR-QTOF-MS and reported to be consistent with natural **87** spectra. Antibiotic evaluations were completed against *S. aureus* and *Pseudomonas aeruginosa*, which showed no antibiotic activity at all concentrations (IC_50_ > 128 μM). Additionally, anti-prolific activity was evaluated against two cancer cell lines: HCT-116 and HepG2. Synthetic **87** showed no significant antitumor activity (IC_50_ > 200 μM). Six analogs of **87** were synthesized through the same route and also lacked biological activity.

**Figure 25:**
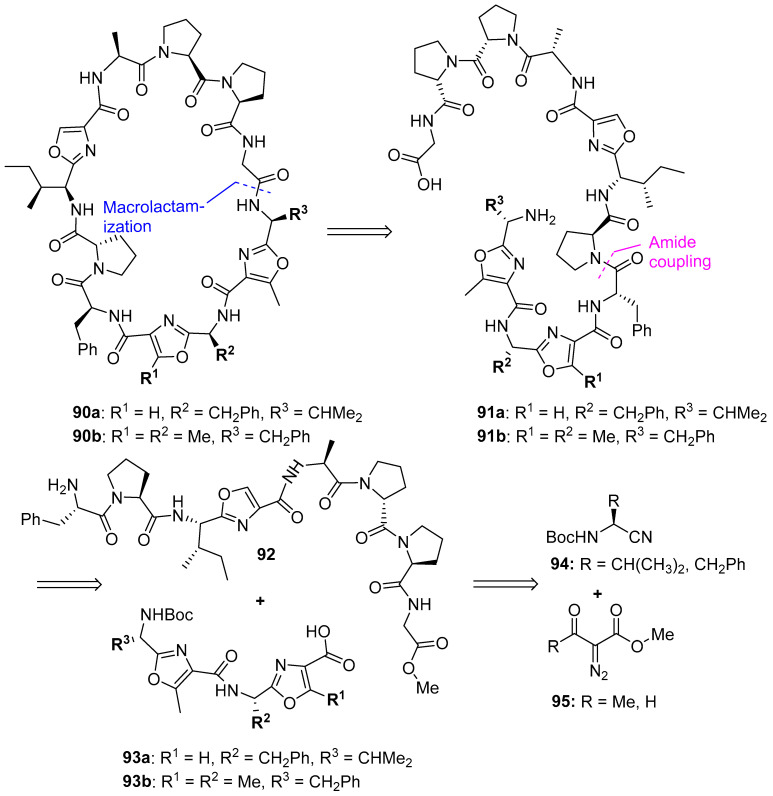
Retrosynthetic analysis of **90a** and **90b**.

Inman et al. [108] aimed to synthesize two natural, cyclic dodecapeptides, wewakazole (**90a**) and wewakazole B (**90b**, Figure 25). Natural **90a** was first isolated from the cyanobacterium *Lyngbya majuscala* in 2003 and showed activity against H-460 (nonsmall cell lung cancer) cells [109]. Natural **90b** was isolated from a different cyanobacterium, *Moorea producens*, roughly 13 years after and reported cytotoxic activity against MCF-7 breast cancer cells (IC_50_ of 0.58 μM) [110]. As the two structures differ in one oxazole moiety and two peptide residues, likewise, they share a common octapeptide sequence. Where **90a** contains 5-unsubstituted oxazole, Phe, and Val, these are replaced with 5-methyloxazole, Ala, and Phe in **90b**, respectively. A convergent process envisioned the common octapeptide fragment coupled accordingly to each bis-oxazole. The oxazole moieties **93a** and **93b** were accomplished with either a rhodium(II)-catalyzed amide N−H insertion of a diazocarbonyl (**95**) for the 5-substituted oxazoles or through a cycloaddition of **95** with nitriles for 5-unsubstituted oxazoles [111]. The linear precursor was constructed through a 4-(4,6-dimethoxy-1,3,5-triazin-2-yl)-4-methyl-morpholinium chloride (DMTMM)-mediated condensation of two peptide intermediates, resulting in the common octapeptide **92**. Cyclization was performed in a head-to-tail orientation using DMTMM at 0.55 mM, giving **90a** in 68% and **90b** in 78% (cyclization) yields. The purified products were compared to the reported literature spectra and matched with no mentioned discrepancies. No biological evaluations were conducted.

**Figure 26:**
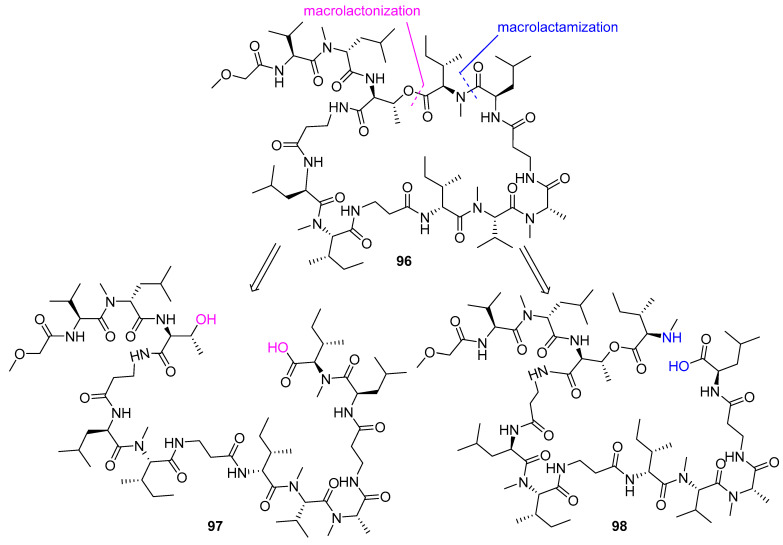
Retrosynthetic analysis of **96**. Macrolactamization versus macrolactonization.

Theonellapeptolides are cyclic tridecadepsipeptides originally isolated from the Okinawan marine sponge *Theonella swinhoei* in the 1980s [112]. In 2000, Roy et al. reported the inhibitory activity of theonellapeptolides Id (**96**, Figure 26), IId, and Ia against mixed lymphocyte reactions (MLRs) and attributed their immunosuppressive behavior as a cytotoxic etiology [113]. Kuranaga and coworkers [114] aimed to synthesize theonellapeptolide Id (**96**, Figure 26) to establish an efficacious synthetic method, confirm its structure, and document its biological activity. Standard SPPS protocol accomplished linear precursor **97**, which underwent head-to-side-chain macrolactonization between D-*allo*-Ile and l-Thr. These conditions promoted the formation of an unwanted diketopiperazine, which was likely due to the *cis*-preferred *N*-methyl amide. A second linear precursor **98** was achieved through SPPS mediated by Oxyma/DIC, tailoring the intermediate for a lactam cyclization between D-*allo*-Ile and D-Leu. Head-to-tail cyclization was optimized with PyBOP/HOAt, obtaining **96** after HPLC purification. NMR characterizations and HPLC retention times were matched with synthetic **96**, with a reported yield of 16.8% (from resin loading, 11 steps). Immunosuppression assays were inherited and repeated as the original studies of Roy et al.: Using RAW 264.7 cell line, osteoclast differentiation was induced by the receptor activator of the nuclear factor-κB ligand (RANKL) [113]. Synthetic **96** showed osteoclast inhibition of 27% at 30 μM, noting 84% cell viability. The authors conclude that **96** is not a potent immunosuppressant, and its activity in vitro is not related to cytotoxicity.

**Figure 27:**
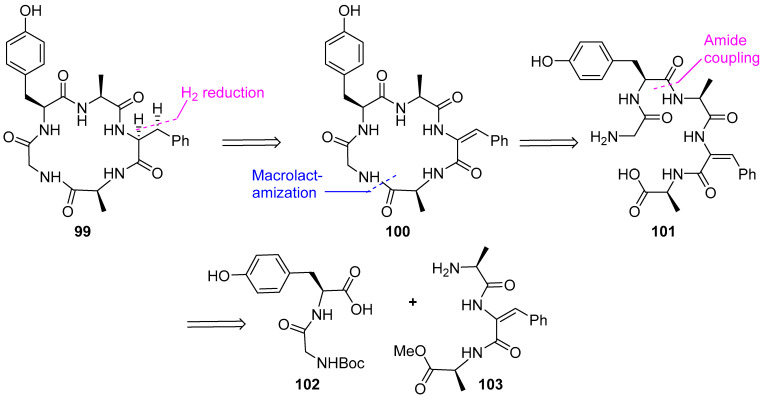
Retrosynthetic analysis of **99**.

Dichotomin E (**99**, Figure 27) is a small, cyclic pentapeptide isolated from the chickweed plant *Stellaria dichotoma*. Following isolation, **99** was established to possess cell growth inhibition against HL-60 leukemia cell lines [115]. The total synthesis of **99** was previously established [116], but Le et al. [117] aimed to improve the cyclization yield by using dehydroamino acids as turn-inducers, followed by catalytic dehydrogenation. This approach was designed to enhance the efficiency of industrial, Gram-scale syntheses of cyclic peptides by circumventing the use of excess solvents under high-diluted conditions. First, the pentapeptide **101** was synthesized in the solution phase using either EDCI/HOBt or HATU/HOAt as the coupling agents from intermediates **102** and **103**. The linear precursor **101** was subject to head-to-tail macrolactamization using HATU/HOAt at 0.1 M or 0.05 M concentrations, resulting in 74% and 81% yields at 0.1 and 0.05 M concentrations, respectively. Under these conditions, **100** gave a monomer:dimer ratio of 20:1 and 39:1, respectively. A saturated linear analog was also synthesized and cyclized under the same conditions. This precursor resulted in a 15% yield of **100** with a 1.5:1 monomer:dimer ratio at 0.1 M. An enantioselective hydrogenation using a Rh(I) catalyst, Rh(cod)_2_BF_4_, and (S,S′,R,R′)-DuanPhos in H_2_ atmosphere (30 atm), resulted in **99** in 95:5 dr. Subsequent NMR studies and CD spectra **101** collectively revealed evidence for preorganized structures resulting from an enamide within the linear precursor. The synthesis exploits an economical method to cyclize linear peptides in up to 100 times more concentrated conditions compared to conventional approaches.

**Figure 28:**
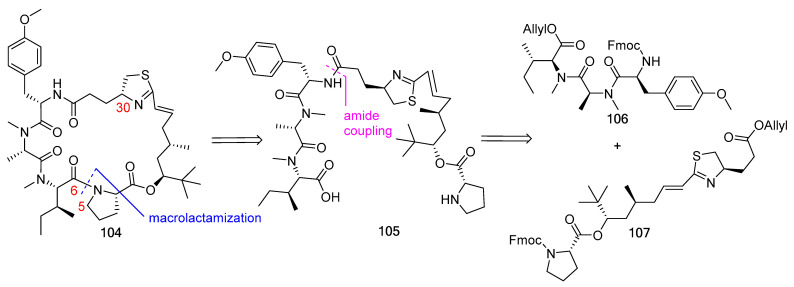
Retrosynthetic analysis of **104**.

Mao et al. [118] explored the total synthesis of the revised structure of apratoxin E (**104**, Figure 28). Natural **104** was also evaluated and synthesized by the Luesch group [119], a species shown to harvest many apratoxins possessing biological activities [120,121,122]. In their original reports, position C30 was assigned *S*-configuration, but subsequent structural intervention revised the absolute configuration to be (*R*)-C30, correctly shown in Figure 28. The synthesis was initiated divergently by utilizing natural starting materials for constructing the linear intermediates **106** and **107**. A chiral lactone from saponin glycoside degradation (industrial waste) and Gln were used to synthesize polyketide fragment **107**, while the separate linear tripeptide **106** was conventionally synthesized through linear amide coupling. These two intermediates were coupled together to obtain the linear precursor **105** for cyclization. Following deprotection, head-to-tail macrolactamization was performed between Ile and Pro residues (C6-N) using HATU as the coupling agent at 5 mM. Cyclization was optimized to recover yields of 21% after semipreparative HPLC. Retention times and NMR characterizations (1D and 2D) were compared to and matched with natural **104** (in addition to m.p. and specific rotation). No biological studies were conducted.

**Figure 29:**
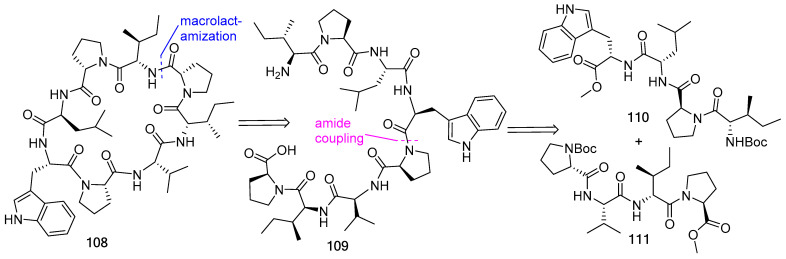
Retrosynthetic analysis of **108**.

Reniochalistatin E (**108**, Figure 29) is one of five related cyclic peptides isolated from the marine sponge *Reniochalina stalagmitis* in 2014 by the Lin group [123]. The reniochalistatin family of peptides is characterized as Pro-rich cyclic heptapeptides containing mostly nonpolar amino acid residues [124,125]. However, **108** is unique among the family, as it is the only octacyclicpeptide member and is the only member containing a Trp residue within its structure. Additionally, preliminary biological results suggested **108** is the most biologically active member, exhibiting in vitro cytotoxicity against myeloma (RPMI-8226, IC_50_ of 4.9 μM) and gastric (MGC-803, IC_50_ of 9.7 μM) cancer cell lines [126], making its synthesis biologically relevant.

Fatino et al. [127] established a solution-phase convergent synthesis of **108**. The two linear precursors, **110** and **111**, were generated through successive solution-phase peptide couplings and eventually coupled, resulting in **109**. Following deprotection, head-to-tail cyclization was performed between Pro and Trp residues, resulting in **108**. Several peptide coupling agents were screened, but EDCI/HOBt (at 1 mM) resulted in the best yields (15%). In vitro, cytotoxicity assays were conducted against myeloma (RPMI-8226) and cervical cancer (HeLa) cells, which showed IC_50_ values of 4.5 μM and 16.9 μM, respectively. These biological results are essentially the same as previously reported for natural **108**.

**Figure 30:**
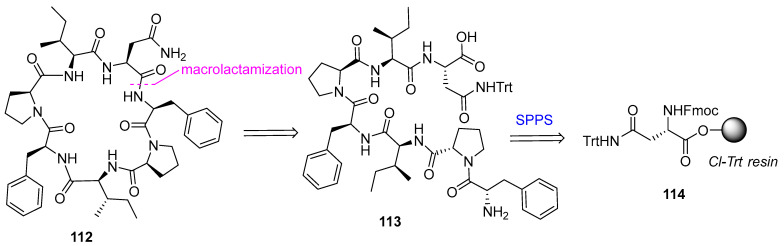
Retrosynthetic analysis of **112**.

Li et al. [128] reported the total synthesis of the cyclic heptapeptide euryjanicins E (**112**, Figure 30), a natural product isolated from the Caribbean sponge *Prosuberites laughlini* [129]. Although naturally harvested **112** showed no activity against cancer cell lines, its close relatives share remarkable antiprolific activities [130]. By application of SAR, however, **112** could be post-synthetically modified to a biologically active analog. Intermediate **114** was achieved by loading protected Asn on a chlorotrityl resin and linearly elaborated to **113** using SPPS in the presence of HCTU/DIPEA in 85.1% yield. Head-to-tail macrolactamization was conducted in solution between Asn and Phe residues in the presence of PyBOP/HOBt using DIPEA/NMP at 0.5 g/mL (linear precursor). The cyclization resulted in **112** in 82.5% yield after chromatography with sephadex LH-20. NMR studies and HR-QTOF-MS confirmed the structure compared to natural **112**. No biological studies were conducted.

**Figure 31:**
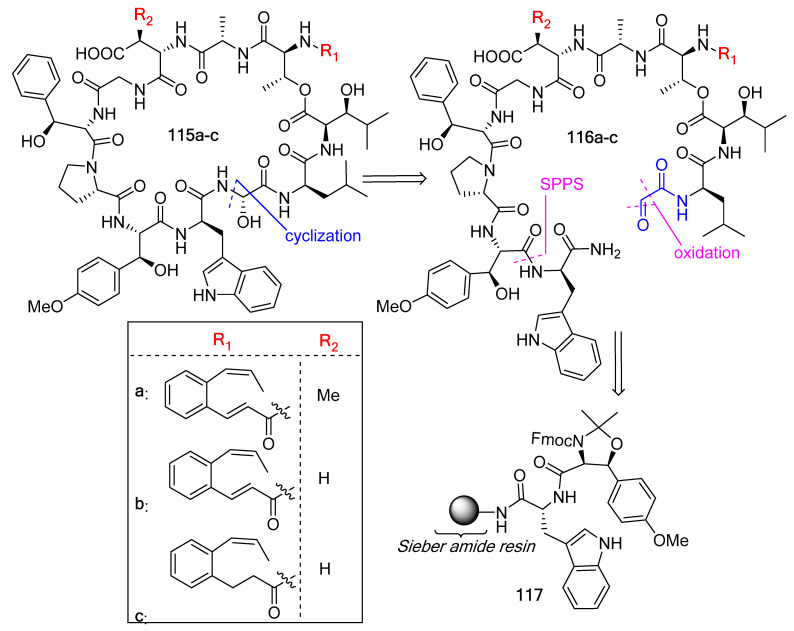
Retrosynthetic analysis of **115a**–**c**.

Skyllamycins are a family of non-ribosomal cyclic depsipeptides produced by *Streptomyces* sp. [131] The family consists of several unique amino acid residues and moieties such as three β-OH amino acids (β-OH-Phe, β-OH-*O*-Me-Tyr, β-OH-Leu), an N-terminal cinnamoyl residue, β-Me-Asp, and an α-OH-Gly residue. The isolated natural products skyllamycin A (**115a**), B (**115b**), and C (**115c**) were found to inhibit *P. aeruginosa* biofilm formation [132]. The Payne group [133] generated a reliable synthesis of compounds **115a-c**, starting with SPPS using a Sieber resin. In the presence of PyAOP, HATU/HOAt, or DIC/DMAP (with or without microwave irradiation), each linear precursor was elaborated successfully in 11–12% yields after HPLC purification. Prior to cyclization, each linear precursor was oxidized at the C-terminal using sodium periodate in a NaHPO_4_ buffer, generating glyoxylamide intermediates (**116**). Intramolecular cyclization was carried out in a head-to-tail manner of each linear glyoxylamide at 1.0 mM in acetonitrile at 60 °C with no coupling agent or catalyst. Notably, cyclization was first carried out under acid-catalyzed conditions (1% TFA) but resulted in products unrelated to **115**. The cyclized products were purified using HPLC methods acquired in 19–33% yields (cyclization). Synthetic **115a**–**c** were tested against *P. aeruginosa* and possessed the same activity as their natural counterparts (skyllamycins A–C) at inhibiting biofilm formation (Figure 31).

**Figure 32:**
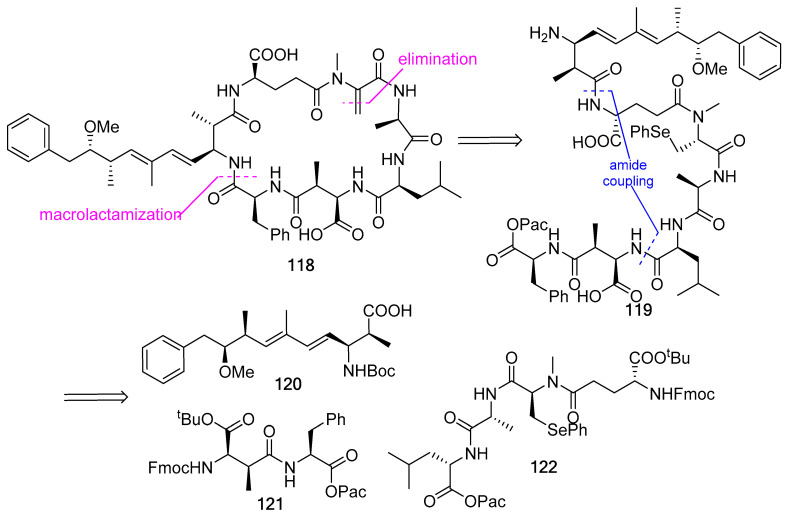
Retrosynthetic analysis of **118**.

Microcystins are produced by various cyanobacteria and are considered dangerous water pollutants affecting densely populated areas [134,135]. Their toxic nature has interested scientists to aid in identifying the cyanotoxins as well as developing analogs relevant to medicine/nature. The Wittmann group reported a solution-phase synthesis of microcystin-LF, **118** (Figure 32), which contains phenyldecadienoic acid and *N*-methyldehydroalanine residues described as vital to their biological function [136,137]. First, the synthesis of three fragments **120**–**122** provided positional handles for accomplishing linear heptapeptide **119** through two convergent amide couplings in the presence of HATU. Following deprotection, **119** was subject to head-to-tail macrolactamization by activating the carboxylic acid with pentafluorophenyl ester and subsequently cyclizing under basic conditions using a two-phased buffer-solvent system (phosphate buffer/CHCl_3_, pH 9.5). In a subsequent selenoxide elimination under oxidative conditions (H_2_O_2_), **118** was recovered in 19% yield from the final two steps following HPLC purification. HRMS and NMR (1D and 2D) confirmed the identity. A phosphatase-1 inhibition assay was conducted to confirm the biological activity of synthetic **118**, showing IC_50_ = 870 pM, compared to natural **118**: IC_50_ = 1.2 nM.

**Figure 33:**
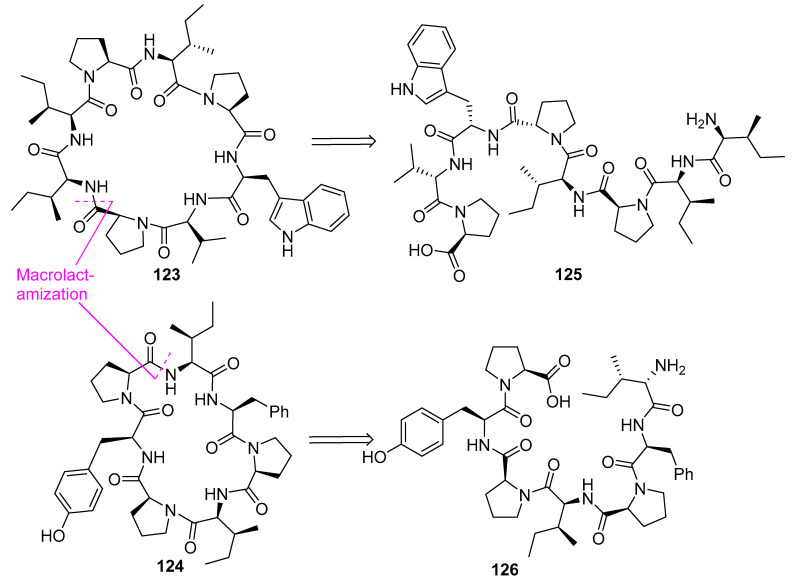
Retrosynthetic analysis of **123** and **124**.

Phakellistatins 17 and 18 (**123** and **124**, Figure 33) were originally isolated from the marine sponge *Phakellia fusca* [138,139]. Natural **123** and **124** are structurally unrelated, differing in size and residue composition, but are similarly Pro-rich. Several members within the phakellistatin family have shown in vitro antiprolific activity against several cancer cell lines [139]. Wu et al. [140] reported the first total synthesis of both **123** and **124** using a solid- and solution-phase hybrid synthesis. Both linear peptides were elaborated separately on a chlorotrityl chloride resin in the presence of HCTU from a common on-resin Pro intermediate. Linear intermediates **125** and **126** were subject to solution-phase macrolactamization in a head-to-tail orientation using PyBOP/HOBt. Overall yields, after semi-preparative HPLC, were reported up to 22.1%. The final products were characterized by NMR studies and HR-QTOF-MS and matched their natural counterparts. MTT cytotoxicity assays were performed on the human lung and liver cell lines, A549 and BEL-7402, respectively. Synthetic **123** showed no cytotoxicity against either cell line, but **124** showed low cytotoxicity: A549 (IC_50_ = 72.42 μM) and BEL-7042 (IC_50_ = 85.25 μM). In addition, two analogs of **124** were generated using the above procedure and conferred improved in vitro cytotoxicity against the same cell lines.

**Figure 34:**
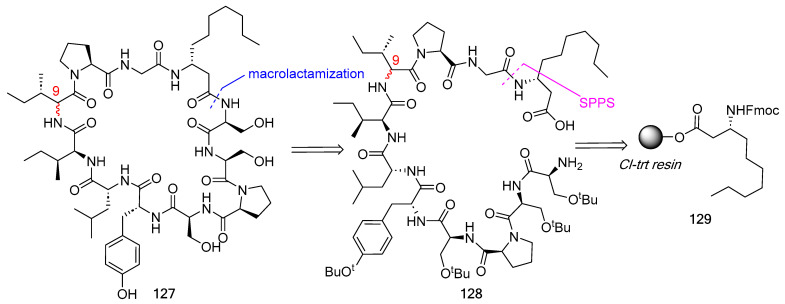
Retrosynthetic analysis of **127**.

Laxaphycins are a large family of cyclic lipopeptides consisting of two subfamilies, LaxA and LaxB, isolated from various types of cyanobacteria. Several family members are proven cytotoxic agents possessing in vitro antiproliferative activity against various cancer cell lines [141,142]. Trichormamide A (**127**, Figure 34) falls within the LaxA subfamily [143] and features three Ser residues, Tyr, and an exocyclic heptanyl chain. Gaillard et al. [144] designed a total synthesis for the proposed structure **127**. The sequence was achieved on a chlorotrityl solid support (**129**) to elaborate linear precursor **128** in the presence of DIC/Oxyma under microwave conditions (50 °C). Macrolactamization was performed using PyOxym/Oxyma between pseudo-high dilution conditions (dual syringe pump) and purified HPLC. HRESI-MS, LC/MS, and NMR studies (1D and 2D) were used for characterization. However, some proton and carbon peaks could not be correctly assigned compared to naturally sourced **127**. According to the mass spectroscopy data, the proposed sequence of **127** was confirmed. No biological studies were performed. Further characterization and isolation of natural **127** will be needed to elucidate and confirm its structure.

**Figure 35:**
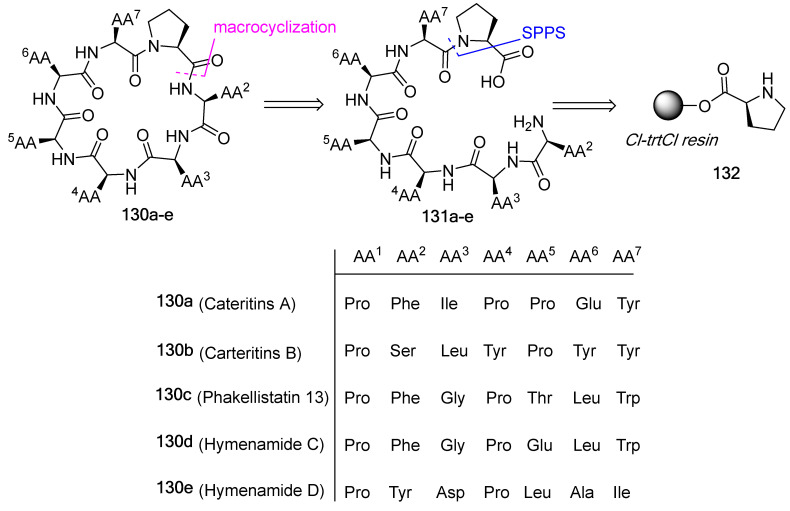
Retrosynthetic analysis of **130a**–**e**.

Chang et al. [145] described a total synthesis method for synthesizing five Pro-rich cyclic peptides isolated from the marine sponge *Stylissa carteri*. The five heptapeptides, carteritin A and B, phakellistatin 13, hymenamide C and D (**130a**–**c**, Figure 35), were isolated in very low yields but exhibited promising cytotoxic activity against several cancer cell lines [146,147,148]. The peptides were constructed by application of SPPS and solution-phase synthesis. On-resin (chlorotrityl chloride) elaboration of each linear analog was completed using HCTU, starting with the on-resin Pro **132**. Once cleaved, head-to-tail macrolactamization of **131a**–**c** was completed in the solution phase using PyBOP/HOBt at roughly 0.5 mg/mL concentrations. Each cyclic peptide was synthesized according to this protocol, with reported yields of ~20% overall. All synthetic products (**130a**–**c**) were evaluated for their cytotoxicities against human cervical cancer (HeLa), human colon cancer (HCT-116), murine macrophage (RAW264.7), human hepatoma (BEL 7402), and human lung carcinoma (A-549) cell lines. All the products showed little cytotoxic activity against the five cell lines with IC_50_ values above 25 μM, despite good spectroscopic structural agreement verified by HR-QTOF-MS and NMR studies.

**Figure 36:**
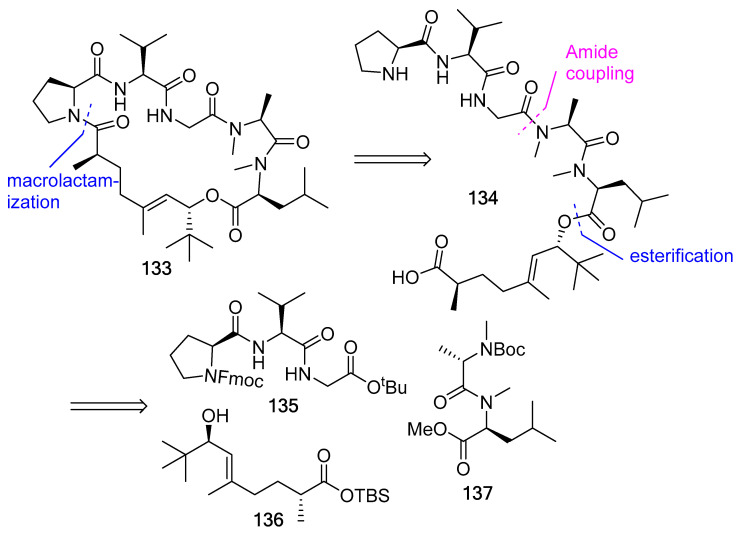
Retrosynthetic analysis of **133**.

The Suenaga group [149] designed a total synthesis of janadolide (**133**, Figure 36), which was isolated in 2016 by the same group [150] when searching for secondary metabolites of cyanobacteria in Okinawa. The isolated natural product showed nanomolar anti-parasitic potency against trypanosomal species, the parasite responsible for African sleeping sickness (IC_50_ = 47 nM) [151]. A straight-forward solution-phase synthesis was employed to achieve linear precursor **134**, using HATU under basic conditions. Intermediate **134** was subject to head-to-tail macrolactamization in the presence of HATU between Pro and the polyketide moiety at 1 mM in DMF. HPLC techniques purified product **133** in 17% cyclization yield. Additionally, macrolactonization was attempted under several conditions but was unsuccessful. Following purification, ^1^H and ^13^C NMR, HRMS, and optical rotation agreed with the spectroscopic data of natural **133**. No biological studies were reported.

**Figure 37:**
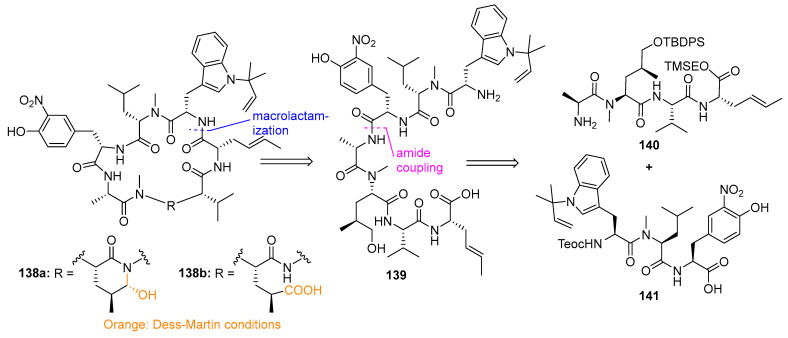
Retrosynthetic analysis of **138a** and **138b**.

Ilamycin E_1_ and F (**138a** and **138b**, Figure 37) are cyclic heptapeptides isolated from the fermentation of marine *Streptomyces atratus* SCSIO ZH16 obtained in 2016 [152]. Only **138a** (Ilamycin E_1_) has been tested against *Mycobacterium tuberculosis* H37Rv.3, showing potent IC_50_ values as low as 9.8 nM [153]. Natural **138a** contains a 3-amino-6-hydroy-2-piperidone (Ahp) moiety, whereas **138b** contains 2-amino-4-methylpentanedioic acid residue. The total synthesis reported by Cheng et al. [154] encompassed a convergent solution-phase protocol. The synthesis of tetrapeptide **140** and tripeptide **141**, in the presence of either EDCI/HOAt or HATU/HOAt, allowed for a convergent formation of the protected linear precursor. Fragments **140** and **141** were coupled using PyAOP and subsequently deprotected, giving intermediate **139**. Once the conditions were optimized, intermediate **139** was cyclized in a head-to-tail orientation using diphenylphosphinic acid pentafluorophenyl ester (FDPP) with DIPEA (yield = 44%, no concentration indicated). Finally, **138a** and **138b** were achieved under separate Dess–Martin conditions. Product **138a** was accomplished using Dess–Martin conditions, yielding an aldehyde that was directly treated with potassium carbonate (K2CO3) in methanol (MeOH), leading to the Ahp moiety in **138a**. Two consecutive oxidations were required for **138b**, starting with the same Dess–Martin conditions, leading to an aldehyde intermediate. This was oxidized to a carboxylic acid under Pinnick conditions, giving **138b**. The authors also reported converting **138b** to **138a** (and visa vera) through two synthetic steps. No biological studies were reported.

**Figure 38:**
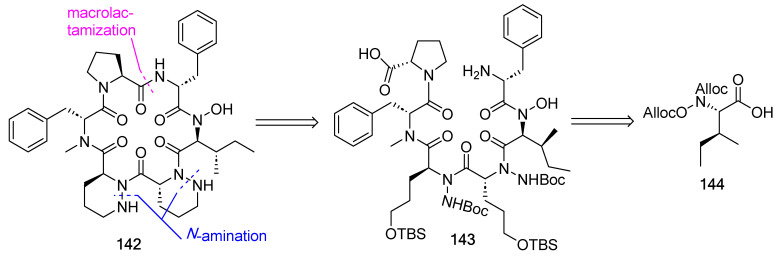
Retrosynthetic analysis of **142**.

L-156,373 (**142**, Figure 38) is a hexapeptide isolated from the fermentation broth of *Strepomyces silvensis*. Using radioligand binding assays, **142** showed a binding affinity (K_i_) of 150 nM for the oxytocin receptor [155]. Additionally, naturally sourced **142** has been synthetically modified, conferring enhanced biological activity toward Arg vasopressin receptor V_2_ [156], but had not been fully synthesized until Elbatrawi et al. [157] designed its first total synthesis. Its unique structure contains two piperazic acid moieties with opposite absolute configurations, one *N*-methylated residue, and four nonpolar residues. The initial challenge resided in synthesizing the protected *N*-hydroxyisoleucine building block **144** from d-*allo*-Ile. Once optimized, the linear precursor was elaborated in the solution phase by consecutive peptide couplings using either HATU or 1-Chloro-*N*,*N*, 2-trimethyl-1-propenylamine (Ghosez’s reagent) under basic conditions, affording **143**. Finally, macrolactamization was achieved by head-to-tail coupling using HATU leading to an 82% yield (concentration not reported). Subsequent tandem intramolecular *N*-aminations under basic conditions formed each piperazic acid residue, resulting in product **142**. Importantly, erosion of product yield in the final step was secondary to the slow elimination of water in the presence of the (*N*-OH)-Ile residue. NMR was used to confirm the structure of **142** to its naturally sourced counterpart, with no noted discrepancies. No biological studies were reported.

**Figure 39:**
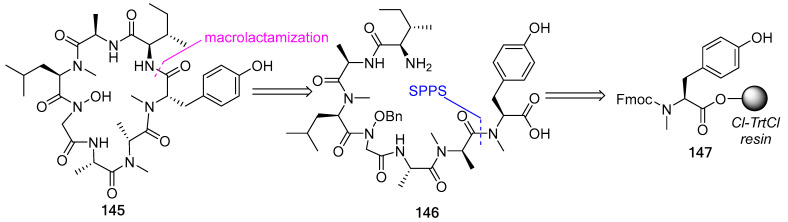
Retrosynthetic analysis of **145**.

The Brimble group [158] designed a second-reported total synthesis of talarolide A (**145**, Figure 39) with enhanced yields and the absence of corrosive reagents. Talarolide A is a heptapeptide isolated from the fungus *Talaromyces* sp. CMB TU011 by the Capon group, who first spectroscopically elucidated its structure [159]. The cyclic peptide is proposed to possess an *N*-hydroxy amide moiety and several *N*-methyl amino acid residues. These structural features have been proposed as inhibitors of *Mycobacterium tuberculosis* and the oxytocin receptor [160,161]. The linear precursor **146** was first elaborated on a chlorotrityl chloride resin in the presence of HATU from **147**. Unpredictably, repeated Fmoc deprotection with piperidine resulted in a thermal reduction in the benzyl-protected (*N*-OH)-Gly to a C-terminal Gly product. The linear precursor was revisited, and it was found that thermal reduction was inhibited when the (*N*-OH)-Gly was placed in the middle of the peptide sequence. Therefore, cyclization between *N*-Me-Tyr and d-*allo*-Ile was required (Figure 39). Following resin cleavage and global deprotection, head-to-tail macroclactamization was performed in the solution phase in the presence of PyBOP at 0.75 mM. From resin loading to cyclization, overall yields were reported at 16%. Cyclization was followed by LC/MS, showing a single peak with the predicted mass of **145**, while HMBC confirmed the sequence. However, ^1^H NMR and ^13^C NMR did not match the reported spectra for natural **145** and suggested a 1:1 ratio of two different conformers. Subsequent 2D NMR studies (i.e., ROESY), along with temperature-dependent NMR, suggested the presence of two conformers: a *cis*/*trans* relationship between D-*N*Me-Leu and d-Ala with a relatively high energy barrier. No biological studies were performed on **145**.

**Figure 40:**
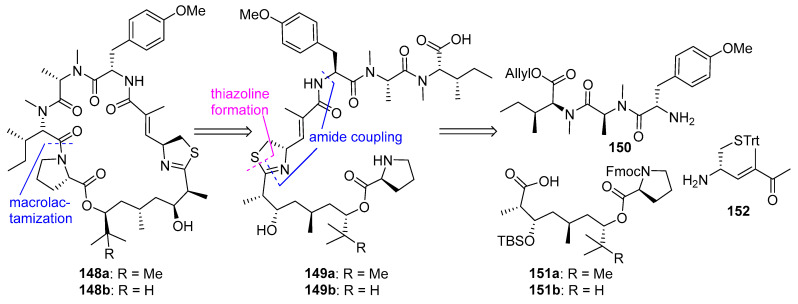
Retrosynthetic analysis of **148a** and **148b**.

Apratoxin A is a cytotoxic natural compound that inhibits the biogenesis of secretory and membrane proteins. It exerts cytotoxic effects by directly blocking the Sec61 protein translocation channel [162]. Reported in 2001 by Luesch et al. was the isolation and characterization of apratoxin A (**148a**, Figure 40), extracted from cyanobacterium *Lyngbya majuscula* Harvey ex Gomont [97]. Computational studies of apratoxins were investigated to understand their reactivity and potential therapeutic application [163]. More recently, Yoshida and co-workers [164] designed a total synthesis of **148a** and **148b**, along with their unnatural oxazoline analogs, and evaluated their biological activities against various cancer cell lines. Both **148a** and **148b** contain a unique dimethylhexanol chain (Dtrina) incorporated into their ring systems, which were synthesized as separate building blocks (**151a** or **151b**). Solution-phase condensation of each **151** fragment with intermediates **150** and **152** led to the respective linear analog (**149a** and **149b**) with the assistance of an eloquent thiazoline ring formation using Kelly’s method. Macrolactamization was completed in the presence of HATU diluted to 1 mM. Furthermore, **148a** and **148b** had reported cyclization yields of 53% and 21%, respectively. Against HCT-116, **148a** and **148b** showed highly potent inhibition with IC_50_ values of 3.2 and 4.1 nM, respectively. Additionally, **148a** was tested against pancreas, stomach, lung, and liver cancer cell lines (among others) with reported growth inhibition 50% (GI_50_) values in the low nanomolar (nM) range, superior to positive control mitomycin C.

**Figure 41:**
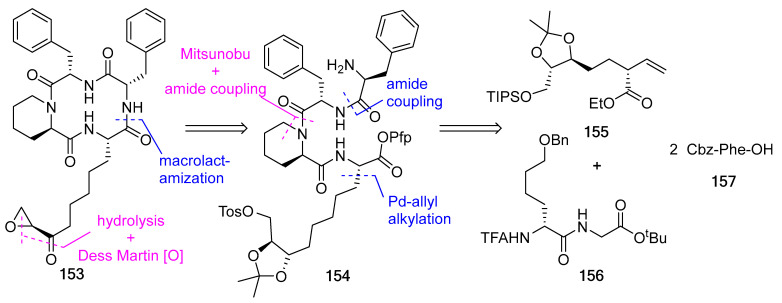
Retrosynthetic analysis of **153**.

Trapoxin A (**153**, Figure 41) is a fungal secondary metabolite from *Helicoma ambiens* RF-102310 with implications as an anticancer agent through antagonistic effects of histone modification enzymes [165]. Servatius and Kazmaier [166] reported the synthesis of the small tetrapeptide containing an exocyclic hexane chain with a terminal α-epoxyketone moiety. Intermediate **155** served as the α-epoxyketone precursor and was achieved through six linear steps. Intermediates **155** and **156** underwent a Pd-catalyzed allylic alkylation; two subsequent amide couplings with **157** afforded the linear precursor in the presence of TBTU. The C-terminal was then activated to **154** by exposure to pentafluorophenol/EDC, and the direct head-to-tail cyclization was achieved under tandem H_2_-mediated cbz-deprotection. However, intermediate **154** was resistant to classical cbz-deprotection conditions, deeming cyclization yields limited by deprotection yields (28%). The synthesis of **153** was completed by ketal acid hydrolysis, base-mediated epoxide formation, and a Dess–Martin periodinane oxidation, achieving the terminal α-epoxyketone moiety. NMR studies matched the natural product’s spectra. No biological studies were reported.

**Figure 42:**
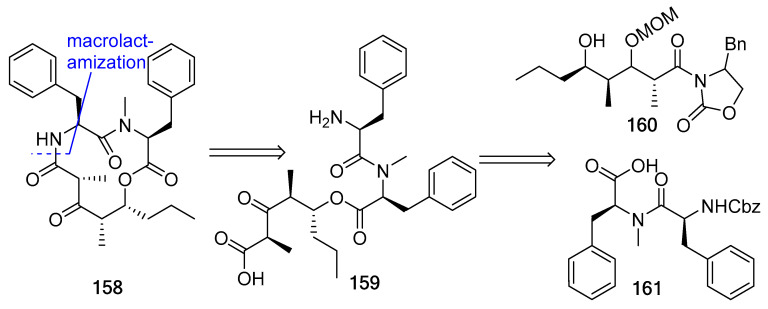
Retrosynthetic analysis of **158**.

Stereocalpin A (**158**, Figure 42) is a small, cyclic dipeptide–polyketide hybrid showing preliminary evidence of antitumor and anti-inflammatory properties [167,168]. Natural **158** was isolated from an Antarctic lichen, *Stereocaulon alpinum*, but the structure was only partially elucidated, and several attempts to synthesize and characterize **158** were unsuccessful [168,169]. A solution-phase total synthesis was achieved by Oishi and co-workers [170]. To begin, the polyketide precursor **160** (protected diol) was synthesized through a three-step transformation of 2-methylpropane-1,3-diol. Fragment **161** was convergently added to **160** through Steglich esterification conditions, giving the protected linear precursor. Linear precursor **159** was cyclized through head-to-tail macrolactamization and accomplished in 54% yield using HATU/HOAt. Subsequent alcohol deprotection and Dess–Martin oxidation resulted in **158**. Extensive characterization with ^1^H/^13^C NMR, 2D NOESY NMR, and HRMS was compared to all stereoisomers synthesized (eight in total) and natural **158**. The C5-methyl at the polyketide moiety could not be fully elucidated based on NOE. However, full characterization determined the polyketide moiety to possess 2*R*,4*S*,5*R*-configuration, correctly shown in Figure 42. No biological studies were performed.

**Figure 43:**
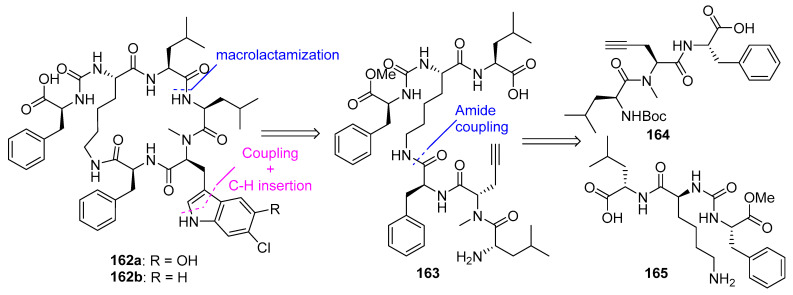
Retrosynthetic analysis of **162**.

Junk and Kazmaier [171] reported the total synthesis of the proposed structures of keramamides A and L, **162a** and **162b** (Figure 43). Natural **162a** and **162b** were first isolated in the 1990s by Kobayashi and co-workers from a *Theonella* sp. marine sponge [172,173]. Once characterized, researchers found that **162a** inhibited a Ca^2+^-ATPase, while **162b** was found to possess moderate cytotoxicity against several cancer cell lines [173]. The peptides are uniquely structured, both categorized as hexapeptides but containing five amino acids within the ring system. The Lys side chain is encompassed within the ring system, leaving an exocyclic Phe residue. Natural **162a** and **162b** differ at position 5 of the indole ring with the presence or absence of a hydroxyl group (R), respectively. A solution-phase convergent synthesis was employed in which tripeptides **164** and **165** were synthesized separately and coupled at the Lys amine R-group to Phe using isobutyl chloroformate (IBCF), resulting in the protected linear precursor. Deprotection of the linear intermediate resulted in **163**, which was subject to macrolactamization, mediated by TBTU at 1 mM in good yields (69%). A post-cyclization cross-coupling and azidation were implemented to allow for the formation of each Trp indole ring. In each case, the synthesis was completed with a photochemical nitrene C–H insertion of the azide moiety, resulting in the respective indole moieties in **162a** and **162b**. Moreover, 1H and 13C NMR characterized each product. It was found the synthesized products were epimers of their natural counterparts at the Lys residue. Replacing the L-Lys with D-Lys (using the same synthetic route) led to characterizations in good agreement with natural **162a** and **162b** spectra. No biological studies were reported.

**Figure 44:**
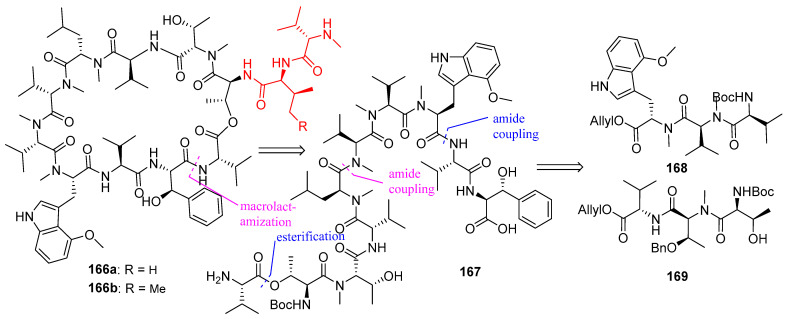
Retrosynthetic analysis of **166a** and **166b**.

Suh and co-workers [174] described a total synthesis of two cyclic depsipeptides, ohmyungsamycins A (**166a**) and B (**166b**). The decapeptides were isolated in 2013 from *Streptomyces* sp. by Oh and Shin, who reported strong preliminary data suggesting their cytotoxic potentials [175]. The peptides contain ten amino acid residues, including four *N*-methylated amides and two nonproteinogenic residues [175]. The two natural products differ in the methylation pattern of the dipeptide side chain (R-group, Figure 44) at position 10, where **166a** is *N*-monomethylated and **166b** is *N*-dimethylated. Peptide fragments **168** and **169** were assembled separately to accomplish a convergent solution-phase synthesis, forming the common linear precursor **167** following deprotection. Intermediate **167** was subjected to macrolactamization in a head-to-tail method. Cyclization conditions were augmented using PyBOP at 0.5 mM, producing good yields after purification (69%). HRMS, 1D, and 2D NMR studies, including COSY and e-HSQC, revealed an agreement with naturally sourced **166a** and **166b**. *M. tuberculosis* assays (positive control, ethambutol MIC_50_ = 3.1 μM) were completed and revealed that **166a** had a potent activity with MIC_50_ = 33.3 nM and **166b** had a lower inhibitory potential (MIC_50_ = 108.3 nM).

**Figure 45:**
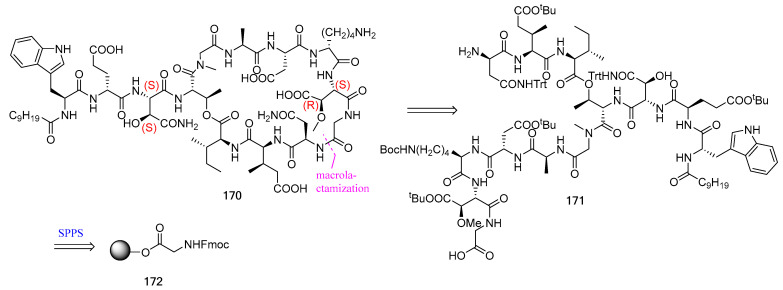
Retrosynthetic analysis of **170**.

Chen et al. [176] reported their total synthesis of the cyclic lipodepsipeptide, A54145 (**170**, Figure 45), isolated from *Streptomyces fradiae* in 1990 [177]. Natural **170** structurally resembles daptomycin and shares potent antibiotic properties against Gram-positive bacteria. Although structurally, **170** was fully characterized, the absolute configuration at two positions was not established [177]. Therefore, each positional epimer was synthesized and spectroscopically compared to a natural sample. The linear peptide was constructed using SPPS on a chlorotrityl chloride resin in the presence of HATU starting with on-resin **172**. Once deprotected, linear analog **171** was cyclized head-to-tail under diluted (5 mM) lactamization conditions using PyBOP. The average overall yield of each **170** diastereomer was reported to be around 3% (from resin loading). NMR analysis was used to compare each synthetic **170** analog to the naturally sourced **170** diastereomers. The corrected absolute configurations at positions 3 and 9 were found to be l-3*S*-HO-Asn and l-3*R*-MeO-Asp, as seen in Figure 45. Synthetic **170** was then tested against three Gram-positive bacterial strains and showed the same activity as daptomycin (MIC_50_ = 0.5–1.0 μg/mL).

**Figure 46:**
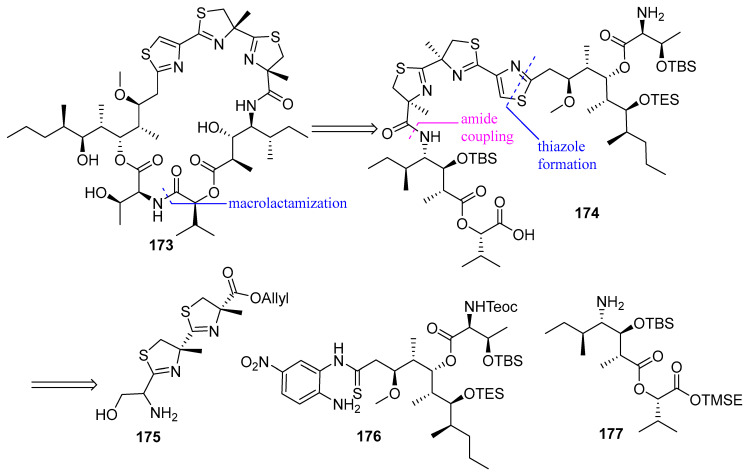
Retrosynthetic analysis of **173**.

Choi, Ye and co-workers [178] established a total synthesis for the neurotoxin hoiamide A (**173**, Figure 46). Natural **173**, isolated from cyanobacteria *Lyngbya majuscula* and *Phormidium gracile* [178], is known as a potent neurotoxin causing neuronal cell death through necrotic and apoptotic pathways [179,180]. Recently, however, **173** was observed to possess agonistic properties toward voltage-gated sodium channels and displayed cytotoxicity against several cancer cell lines [181]. Natural **173** features a 14-carbon polyketide moiety at C30 and three consecutive heterocyclic rings (two thiazoline rings and one thiazole ring). In a convergent process, the synthesis was completed in the solution phase, initiated by constructing fragments **175**–**177**. The *bis*-thiazoline amino acid (**175**) and the linear polyketide fragment (**176**) were combined using a DAST dehydration followed by DBU/CBrCl_3_-mediated oxidation, forming the thiazole ring. Finally, modified Ile fragment **177** was appended by pyridinium-activated esterification. After deprotection, linear intermediate **174** was subjected to HATU/HOAt-mediated head-to-tail macrolactamization at 0.5 mM. Cyclization was reported at 37% yield and ~7% overall. NMR characterizations of synthetic **173** were reported in agreement with its natural counterpart. No biological studies were performed.

**Figure 47:**
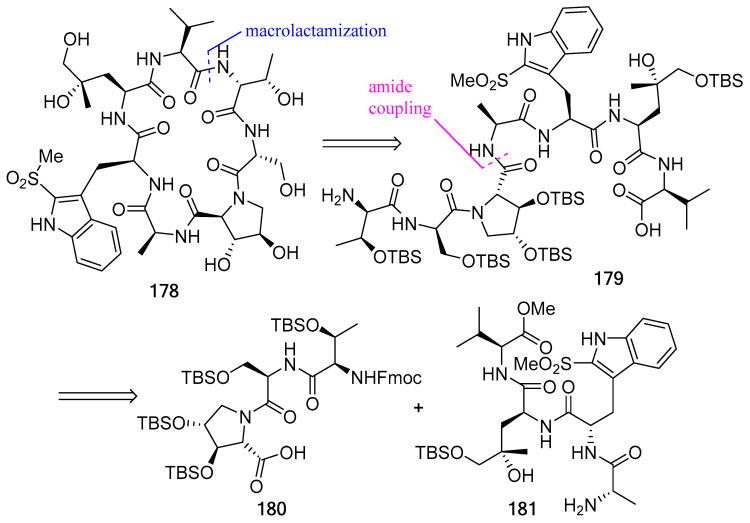
Retrosynthetic analysis of **178**.

Alloviroidin (**178**, Figure 47) is a heptapeptide member of the virotoxin family. It was first isolated from *Amanita virosa* in 1980 by the group of Wieland [182] and later reported to have an LD_50_ of 1–2 nmol/g and possess a strong binding affinity for actin [183]. Taylor and Kutty [184] reported the first total synthesis of **178** and described a convenient solution-phase convergent strategy. The assembly of two peptide fragments, a tripeptide (**180**) and a tetrapeptide (**181**), allowed for the formation of a protected linear precursor following a HATU-mediated coupling between fragments. Deprotection resulted in linear intermediate **179** in good yield (80%), which was cyclized in a head-to-tail fashion using HATU at 1 mM (optimized using DCM). The cyclized product was obtained in yields of 84% and subsequently underwent global deprotection to give **178**. The overall yield, however, was eroded under the final deprotection conditions despite circumventing efforts. The 1H and 13C NMR characterized synthetic **178** and were in agreement with natural alloviroidin spectra. No biological evaluations were reported.

**Figure 48:**
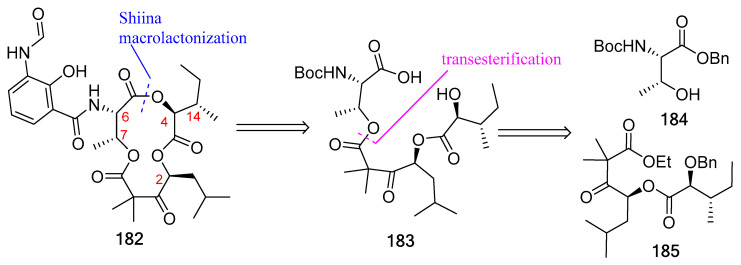
Retrosynthetic analysis of **182**.

JBIR-06 (**182,** Figure 48), a recent member of the antimycin family, was isolated from *Streptomyces* sp. ML-55 by Shin-ya and co-workers [185]. Subsequent studies found that **182** decreased the expression of glucose-regulated protein 78 (GRP78), a protein upregulated during stages of carcinogenesis, in the epithelial HT1080 cells (IC_50_ = 262 nM) [186]. Antimycins vary in ring sizes, but all feature an exocyclic 3-(formylamino)-2-hydroxybenzoic acid moiety with an amide linkage to l-Thr. Natural **182** is a 12-membered trilactone containing five stereogenic centers (C2, C4, C6, C7, and C14). Hitherto, only the absolute configuration of the Thr residue (C6) had been confirmed [185]. Hamada et al. [187] reported the first total synthesis of **182** to confirm its structure and determine its stereochemistry. A single fragment was assembled first and combined with *N*Boc-l-Thr to give the linear precursor. Head-to-tail macrolactonization was completed under Shiina conditions, NMBA (2-methyl-6-nitrobenzoic acid)/DMAP, at 1.6 mM. Optimized conditions resulted in yields up to 74% for cyclization. NMR spectra of synthetic and natural **182** were found to be in good agreement. The specific rotation of synthetic **182** was performed and found [α]_D_ −27.5, *c* 0.03, MeOH (Natural **182**: [α]_D_ −30.0, *c* 0.04, MeOH). No biological evaluation was completed.

**Figure 49:**
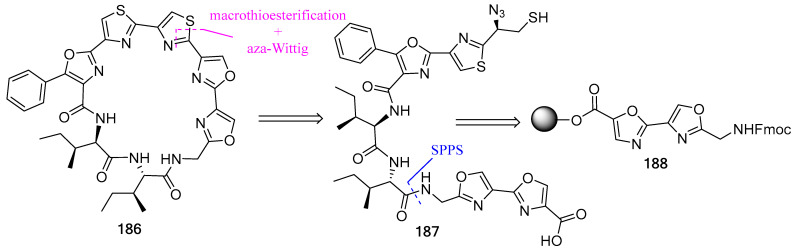
Retrosynthetic analysis of **186**.

Aurantizolicin (**186**, Figure 49) was isolated from *Streptomyces aurantiacus* in 2016 but was not biologically evaluated [188]. Its origin family, the YM-216391 family, features distinct polyazole ring systems and has shown potent cytotoxicity against various cancer cell lines [188,189]. Therefore, Oberheide et al. [190] sought to establish a reliable total synthesis of **186** to confirm its structure and stereochemical assignments. Fmoc-protected SPPS (chlorotrityl chloride resin) was utilized to elaborate the linear tripeptide **187** from one biazole fragment **188**. The coupling reactions during SPPS were mediated by HBTU/HOBt and resulted in a 70% yield of **187**. Following resin cleavage, head-to-tail macrothiolactonization was carried out in the presence of PyBOP at 2 mM, reporting a cyclization yield of 80% under optimized conditions. The final step to achieve **186** involved an *aza*-wittig reaction of the azidothiolactone, resulting in the central thiazoline ring following oxidation with DBU/BrCCl_3_. NMR data, coupled with HPLC and LC-MS, confirmed the structure of synthetic **186** to be identical to the naturally sourced product. Additionally, both diastereomers were synthesized using the same route described and were used for characterization comparison, further reinforcing the structure of **186**. No biological studies were performed.

**Figure 50:**
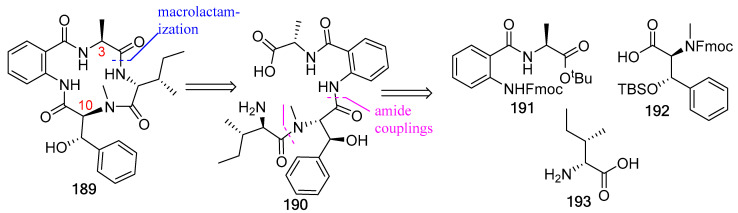
Retrosynthetic analysis of **189**.

Oshawa et al. [191] reported the total synthesis and structural revision of asperterrestide A (**189**, Figure 50), a natural cyclic peptide isolated from the marine-derived fungal strain *Aspergillus terreus* SCSGAF0162 in 2013 [192]. Natural **189** has exhibited cytotoxicity against certain cancer cell lines as well as inhibiting influenza viral strains, H1N1 and H3N2. Stereochemical assignments were completed for all stereogenic centers within the structure except for position 13. Amino acid fragment **191** was synthesized and combined with intermediates **192** and **193,** resulting in a protected linear precursor. Linear precursor **190** was subject to HATU-mediated head-to-tail macrolactamization at 1 mM, resulting in a 41% yield. ^1^H and ^13^C NMR studies, along with 2D NMR data, confirmed the structure and stereochemical assignments of **189** to match its natural counterpart. The cytotoxic evaluation was performed on three cancer cell lines (U937 (lymphoma), Molt4, and A549), showing IC_50_ values of 5.6, 18.1, and >20 μM, respectively.

**Figure 51:**
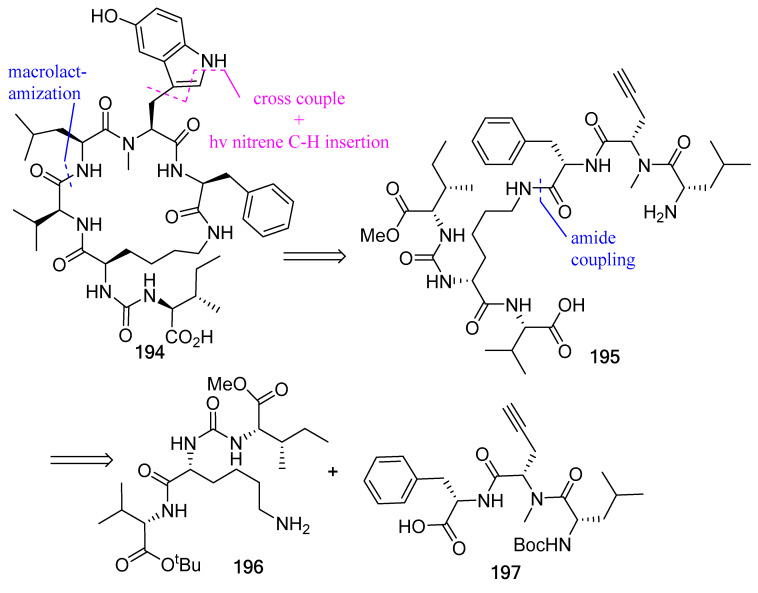
Retrosynthetic analysis of **194**.

Mozamide A (**194**, Figure 51) is a natural cyclic hexapeptide first isolated from the marine sponge of genus *Theonella* in 1997 by Faulkner and co-workers [193]. NMR and MS elucidated the structure, while acid degradation and chiral GCs confirmed its stereochemical assignments. Due to a lack of materials, the biological properties of **194** have not been evaluated, but the anabaenopeptin family shares a range of interesting biological properties [193]. The structure of **194** contains a pentapeptide ring structure connected by the ε-nitrogen of d-Lys, with an exocyclic l-Ile linked by a ureido bond (α-nitrogen, d-Lys). Constructing peptide fragments **196** and **197** allowed for a convergent amide coupling between the d-Lys ε-nitrogen in **196** and the Phe-COOH in **197**. A protected linear precursor resulted from the use of a mixed anhydride activation of **197**. After deprotection, hexapeptide **195** was cyclized using TBTU at 1mM to give a head-to-tail cyclic intermediate in 73% yield. Post-cyclization modifications included a cross-coupling reaction followed by a photochemical nitrene C–H insertion to give **194** after global deprotection. NMR correlations were not in agreement with previously reported data for natural **194**. Two other diastereomers were synthesized, and NMR correlations for one diastereomer were in good agreement (**194**, Figure 51). However, these findings could not be corroborated with an authentic NMR of natural **194**, only what was provided in the literature. It was also noted that **194** was found to be a hydroxylated version of brunsvicamide A, implicating its synthesis by a symbiotic bacteria/fungus within the *Theonella* marine sponge. No biological evaluations were completed [194].

**Figure 52:**
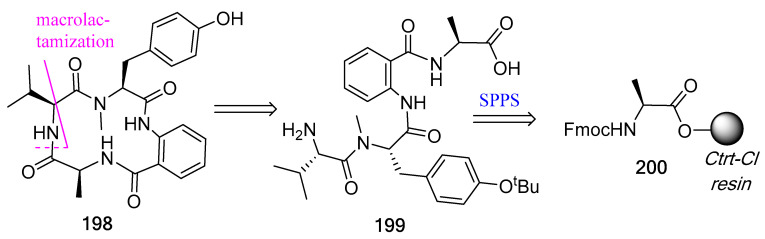
Retrosynthetic analysis of **198**.

In a study by Fukuda et al. [195], nectriatide (**198**) was isolated from a culture broth of *Nectriacae* sp. BF-0114 and was identified as a possible potentiator of amphotericin B (AmB). After structural elucidation, the authors sought to confirm the structure and stereochemical assignments by employing a total synthesis of **198** and its common stereoisomers. The natural product was found to be a tetrapeptide containing an *N*-methyl-l-Tyr, anthranilic acid, l-Ala, and l-Val. Starting with on-resin intermediate **200**Linear precursor, **199** was conveniently elaborated on a chlorotrityl chloride in the presence of Bromo-tris-pyrrolidino-phosphonium hexafluorophosphate (PyBrop) or DIC/HOBt as the activating agent(s). Head-to-tail macrolactamization was accomplished using HATU at 5 mM, resulting in 40–80% yields of each target epimer following HPLC purification. Correlated NMR studies found synthetic and natural **198** to possess the stereochemistry of the isomer shown in Figure 52. Antimicrobial studies showed that the cyclic peptide strongly potentiated AmB activity against *C. albicans* and *Saccharomyces cerevisiae* (up to 16-fold), moderately against *R. oryzae*, and weakly against *A. niger*.

**Figure 53:**
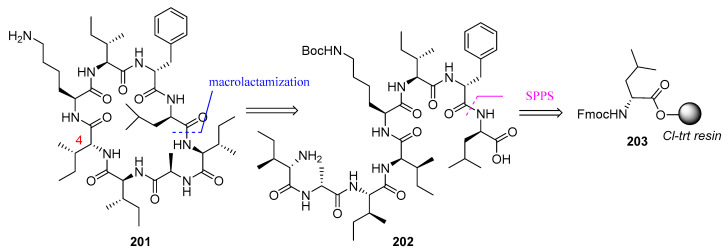
Retrosynthetic analysis of **201**.

Matsuda et al. [196] reported the synthesis of the revised structure of surugamide A (**201**, Figure 53). Natural **201** was first isolated from *Streptomyces* sp. by Takada et al. in 2013, when its inhibitory activity against cathepsin B was also reported [197]. The structure was characterized by 1D and 2D NMR, and stereochemical assignments were identified using Marfrey’s method. However, the absolute configuration at position 4 could not be deciphered as d-Ile or d-*allo*-Ile. The linear octapeptide was assembled by Fmoc SPPS using a 2-chlorotrityl resin in the presence of DIC/Oxyma. Cleavage and deprotection resulted in **202**, which was directly subjected to PyBOP/HOAt-mediated head-to-tail cyclization (c = 4 mM). Following deprotection and HPLC purification (reported 46% yield, 18 steps), the stereochemistry and structural integrity were confirmed using a variety of comparable spectroscopy and Marfrey’s method. No biological studies were conducted.

**Figure 54:**
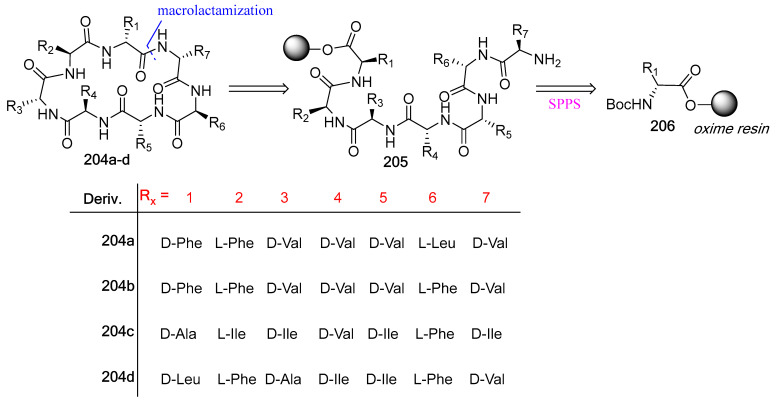
Retrosynthetic analysis of **204a**–**d**.

Reported in 2017, Kerr and co-workers isolated and characterized four cyclic heptapeptides isolated from a *Mortierella* sp., now recognized as mortiamides [198,199]. This group of cyclic peptides contains nonpolar amino acids in a mixture of d-and l-configurations. Bérubé et al. [200] reported the total syntheses of mortiamides A–D (**204a**–**d**, respectively, Figure 54) and presented a unique on-resin cyclization requiring SPPS on an oxime resin. Natural **204a–d** are similar by way of containing only hydrophobic amino acid residues but differ in their identity and structural position. Oligomerization was carried out on an oxime resin (**206**) in the presence of 6-Cl-HOBt and HCTU. Each on-resin linear peptide **205** was simultaneously cleaved and cyclized (head-to-tail) in the presence of buffered DIPEA with acetic acid (1:2 mol/mol) at 0.1 M. This led to reported yields of 35% to 48%. Mortiamides A and B (**204a** and **204b**, respectively) showed moderate activity against *P. falciparum* proliferation, while D (**204d**) exhibited improved, low micromolar activity.

**Figure 55:**
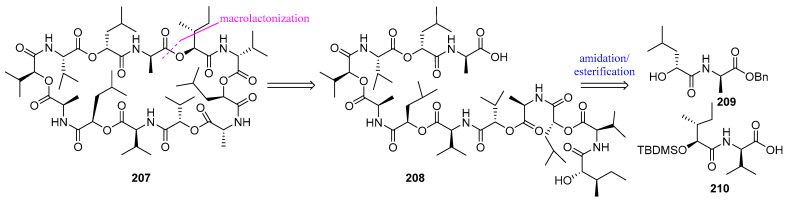
Retrosynthetic analysis of **207**.

Homocereulide (**207**, Figure 55) is a natural product isolated from the marine bacterium *B. cereus* [201]. Preliminary biological results suggest that it possesses similar properties to its analog cereulide [202], a cytotoxic cyclic peptide differing at one amino acid residue with known food poisoning capabilities. Naka et al. [203] devised a synthesis for **207** and sought an evaluation of its biological activity in the context of food poisoning assays (HEp-2 vacuolation). The synthesis was completed in the solution phase and started with linear elaboration of the peptide backbone. Two of the fragments, **209** and **210**, were repeated within **207**, which allowed for a simple divergent strategy. Head-to-tail macrolactonization of **208** was completed under diluted conditions (1.5 mM) using *m*-nitrobenzoic anhydride (MNBAN) and DMAP, obtaining **207** in 11% overall yield (74% cyclization yield). Toxicity was tested by examining vacuole responses in HEp-2 cells (laryngeal cancer). The authors found **207** to be more toxic compared to cereulide, showing vacuolation activity at 1.39 nM, while cereulide exhibited 3.95 nM.

**Figure 56:**
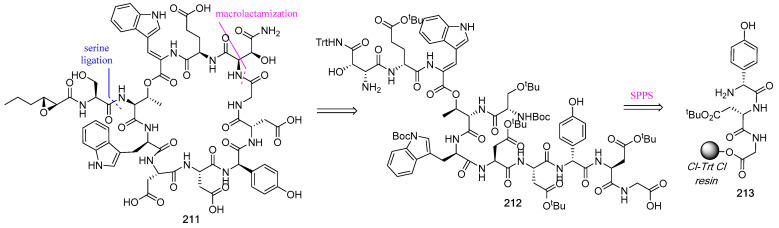
Retrosynthetic analysis of **211**.

CDA3a (**211**, Figure 56) is a calcium-dependent antibiotic (CDA) whose structure was elucidated in the 1980s from a *Streptomyces coelicolor* A3 extract [204]. Nine family members have been identified as CDBs; among these members, CDA3a (**211**) showed the most promising antibacterial properties [205]. Chen et al. [206] reported the first total synthesis of **211** and evaluated its antibacterial properties. The peptide backbone was assembled using Fmoc-SPPS starting with **213** on a chlorotrityl chloride resin in the presence of HATU. Resin cleavage afforded linear precursor **112**, cyclized head-to-tail under lactamization conditions using PyBOP/HOAt at 5 mM (cyclization yield not reported). Finally, an exocyclic fatty acid epoxyhexanoyl salicylic aldehyde was appended with a *N,O*-benzylidene acetal ligation strategy. Respectably, **211** was afforded a 9.3% overall yield after HPLC purification. Synthetic **211** matched natural CDA3a spectroscopically after purification. Minimal inhibitory concentrations were assessed for Gram-positive bacterial strains (MRSA, *S. aureus*, *E. faecalis*, VRE) and showed poor antibiotic activity against all strains. Importantly, several analogs were synthesized and assessed by varying the length of the exocyclic fatty acid chain and showed improved to potent antibacterial activity compared to synthetic **211**.

**Figure 57:**
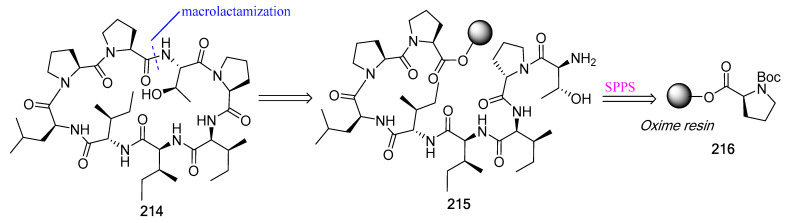
Retrosynthetic analysis of **214**.

In 2005, cyclic octapeptide dominicin (**214**, Figure 57) was isolated from the marine sponge *Eurypon laughlini* by Andersen [207], but no biological testing was reported. Bérubé et al. [208] reported the first total synthesis and biological evaluation of **214**, as it possessed structural characteristics similar to the plant-derived orbitides (antimalarial activity). The octapeptide was elongated on an oxime (Kaiser) resin in the presence of either HCTU/6-Cl-HOBt or HATU/HOAt from intermediate **216**. Initially, the concomitant head-to-tail cyclization–cleavage reaction of **215** was carried out in the presence of dilute (CH_2_Cl_2_) DIPEA/AcOH but resulted in a 7% yield. The authors reasoned that this was secondary to nonpolar aggregations. The conditions were optimized to achieve a 58% cyclization–cleavage yield using DIPEA in the presence of LiBr (5 eq.) at 10^−2^ M. Spectral data and HMBC were in good correlation to natural **214**, with reported overall yields of 30%. The authors reported antimalarial studies of synthetic **214** against two *P. falciparum* strains, 3D7 and Dd2, with IC_50_ values of 3.6 ± 0.2 μM and 1.8 ± 0.2 μM, while the latter strain was highly resistant.

**Figure 58:**
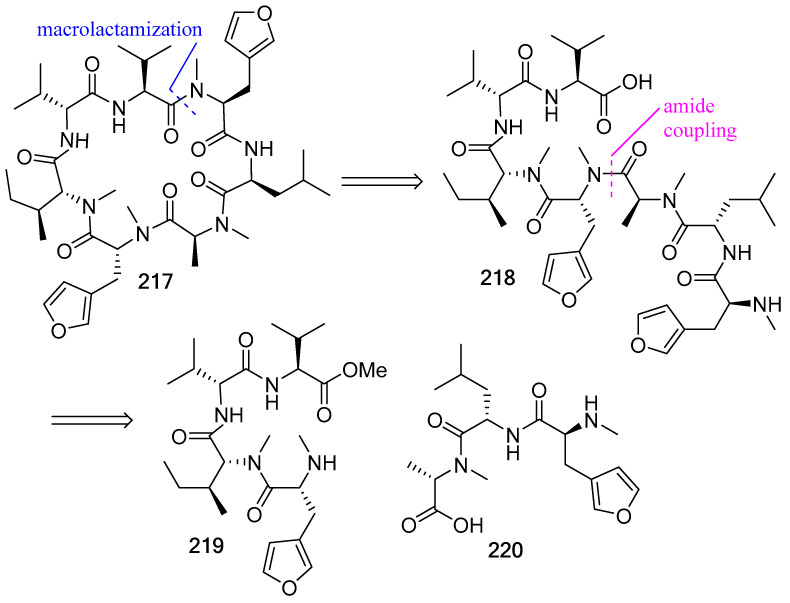
Retrosynthetic analysis of **217**.

Liu et al. [209] reported a total synthesis of rhizonin A (**217**, Figure 58), a non-ribosomal cyclic heptapeptide isolated from the metabolite of *Rhizopus microsporous* originating from *Burkholderia* sp. KCTC 11096 [210]. Preliminary studies with natural **217** indicated that it possessed potent and non-specific hepatotoxic effects [211]. The synthesis was carried out in the solution phase, starting with the convergent construction of tetrapeptide **219** and tripeptide **220**. These two intermediates were coupled using HATU/HOAt-mediated conditions, giving linear precursor **218** following deprotection. Head-to-tail macrolactamization made use of HATU/HOAt as the coupling agent at 1 mM, resulting in **217** in 29% yield. NMR studies and optical rotation were correlative with natural **217**. The authors tested **217** in hypoxia-inducible factor (HIF)-dependent HCT116 colorectal cells and compared the effects of the parental and knockout cell lines. Synthetic **217** preferentially reduced cell viability of HIF and oncogenic (Kirsten Rat Sarcoma Virus) KRAS-containing parental cells compared to the HIF transcription factor-depleted cell lines.

**Figure 59:**
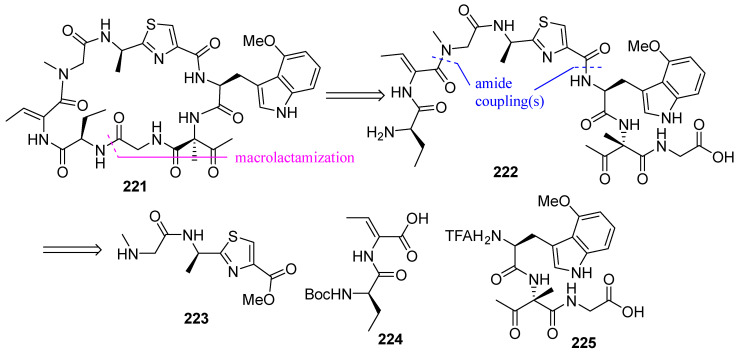
Retrosynthetic analysis of **221**.

Krahn et al. [212] reported the first complete synthesis of zelkovamycin (**221**, Figure 59), a natural cyclic heptapeptide first isolated from the fermentation broth of *Streptomyces* sp. K96-0670 in 1999 by the group of Omura [213,214]. Convergent synthesis of three small peptide fragments (**223**–**225**) was completed in the solution phase and combined in the presence of EDC/HOBt. Head-to-tail macrolactamization was completed in the presence of EDC/HOBt with a respectable yield of 59%. NMR comparisons and MS co-injection studies confirmed the structure of synthetic **221** to be the same as its natural counterpart. The bioactivity of **221** was also reported in a HeLa cell culture, in which the authors concluded the addition of **221** induced a metabolic switch in the cells toward a glycolytic phenotype. Studies indicated that long-term **221** treatment led to an impairment in mitochondrial function.

**Figure 60:**
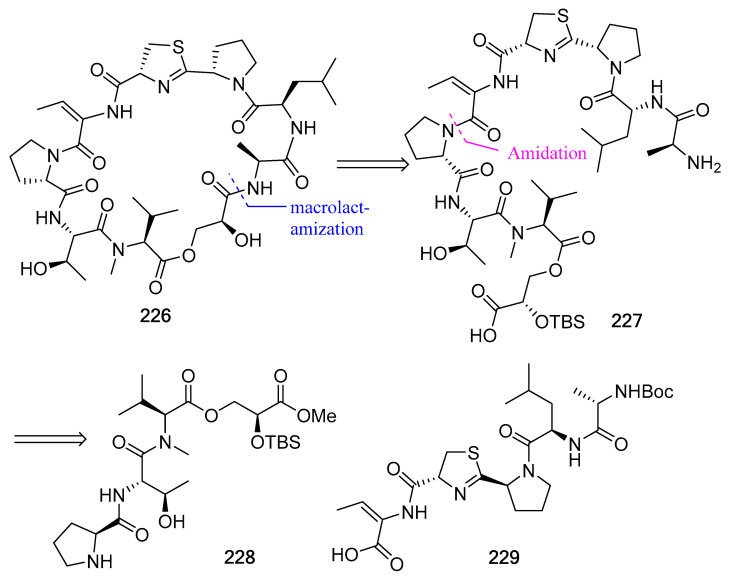
Retrosynthetic analysis of **229**.

Vioprolide D (**226** Figure 60) was first isolated by Schummer et al. from myxobacterium *Cystobacter violaceus* Cb vi35 [215]. Grab et al. [216] reported the total synthesis of the cyclic octapeptide, containing two Pro and a heterocyclic thiazoline moiety. Two linear tetrapeptides, **228** and **229**, were synthesized in the solution phase and coupled together, forming the linear precursor **227** after deprotection. A head-to-tail macrolactamization was accomplished using HATU, resulting in a respectable 40% yield. The dehydrobutyrine residue underwent an inversion reaction (from *Z* to *E*) following cyclization, completing the synthesis. NMR studies (1D and 2D), HPLC, and optical rotation matched the natural **226**. The authors reported an IC_50_ of 679 nM in an MTT assay against acute lymphoblastic leukemia cells.

**Figure 61:**
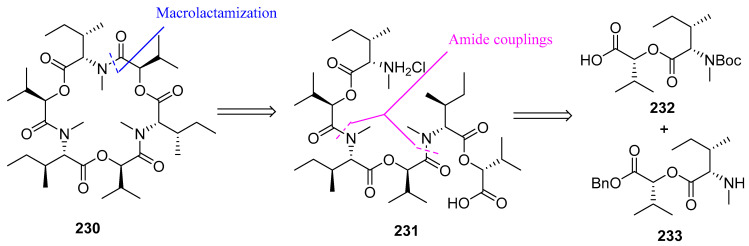
Retrosynthetic analysis of **230**.

Enniatins are cyclic hexadepsipeptides isolated from the fungi genus of *Fusarium* [217] that exhibit fascinating anticancer properties. Previous studies on the enniatins uncovered potent cytostatic or cytotoxic activity against a variety of human cancers via p53-dependent and p53-independent pathways, respectively [218]. Enniatins are also established as inhibitors of the pleiotropic drug resistance 5 protein, an efflux protein responsible for drug resistance in some fungi [219,220]. In this study, the groups of Chadli and Blagg reported the total synthesis of enniatin A (**230**, Figure 61)) and its biological characterization in the context of heat shock protein 90 (Hsp90) inhibition as an important target for the disruption and eradication of triple-negative breast cancer [221,222]. Natural **230** is a cyclic hexamer composed of three repeating oxa–d-valyl-*N*-methyl-L-isoleucyl dimeric subunits. The synthesis of **230** was adopted from a previously reported liquid-phase total synthesis of enniatin B [223]. The repeating dipeptide motif was synthesized, then independently deprotected, producing two key intermediates, **232** and **233**, as the respective carboxylic acid and amine moieties for each consecutive amide coupling mediated by Ghosez’s reagent. Subsequent Boc and benzyl deprotections yielded the linear precursor, **231**, and subject to head-to-tail macrolactamization, again in the presence of Ghosez’s reagent and Hunig’s base (at 5 mM, DCM). Cyclization yields for synthetic **230** were reported to be up to 69% (up to 82% for the synthesized unnatural derivatives), and the characterizations were reported to match those of natural **230** (^13^C/^1^H NMR and mass spectroscopy). In vitro MTT assays using Hs578T breast cancer cells confirmed the cytotoxic properties of synthetic **230** with a reported IC_50_ of 7.39 μM. Further evaluation in vivo revealed significant tumor growth inhibition in E0771-innoculated mouse models with a treatment regimine of 10 mg/kg (*Quaque altera die*, 3 weeks) compared to a DMSO negative control model. Synthetic **230** even outperformed the most potent unnatural in vitro derivative, determined by MTT assays, in live in vivo tumor mouse models after 3 weeks. Further mechanistic studies were investigated using western blot analyses, revealing vast disruption of normal cancer signaling and enhanced elicitation of antitumorigenic signaling upon treatment with **230**. Several derivatives were synthesized in this report (not depicted in Figure 61) for SAR studies, while only **230** showed potent antitumor activity in vitro and in vivo, underscoring the significance of the specific three-dimensional stereochemical requisite for its potent biological activity.

**Figure 62:**
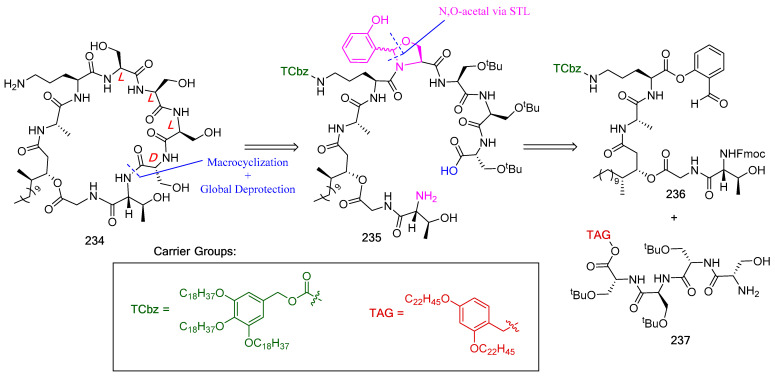
Retrosynthetic Analysis of Tetraselide, **234**.

Tetraselide (**234**, Figure 62) is a polar depsipeptide isolated from a fungal culture broth of *Trichoderma* sp. by the Sunazuka and Hirose groups. Characterization with NMR and LC-MS/MS revealed a cyclic octapeptide featuring a 3:1 ratio of polar to non-polar amino acid residues (Orn, Thr, Ser, Ser, Ser, Ser, Ala, and Gly) and a β-hydroxy-γ-methyl fatty acid moiety. Modified Marfey’s method assisted in identifying L-Ala, L-Orn, D-*allo*-Thr, three L-Ser residues, and one D-Ser residue. However, the location of the D-isomer was ambiguous, as the four Ser residues are found in consecutive sequences within the peptide backbone. The stereochemistry of the β-hydroxy-γ-methyl fatty acid moiety was realized through bioinformatic data and analyzing products of the polyketide synthase pathway specific to *Trichoderma*. These data revealed strong evidence, suggesting a D-β-hydroxyl-D-methyl configuration at the two stereocenters in the fatty acid residue. However, the four Ser stereocenters were still unknown, thus requiring the synthesis and characterization of eight possible stereoisomers. Convergent, liquid-phase synthesis was employed to acquire two peptide fragments, **236** and **237**, each featuring a hydrophobic carrier group (TCbz and TAG, respectively, Figure 62), allowing for intermediate precipitation in polar solvents (e.g., acetonitrile) and uncomplicated isolation. The carrier-supported, protected precursor **235** was quantitatively achieved through Ser/Thr ligation (STL) of intermediates **236** and **237**, optimized using pyridine/AcOH, and resulted in **235** bearing an *N,O*-acetal moiety (pink, Figure 62) as a diasteromixture. Head-to-tail macrolactamization was achieved using EDCI and HOBt in the presence of DIPEA in chloroform (concentration not reported), giving **234** in 9% yield (final 4 steps) after deprotection. The authors confirmed the structure spectroscopically compared to naturally sourced **234**, revealing the four consecutive Ser residues oriented in L-L-L-D configurations. No biological studies were conducted on natural or synthetic **234** [224].

**Figure 63:**
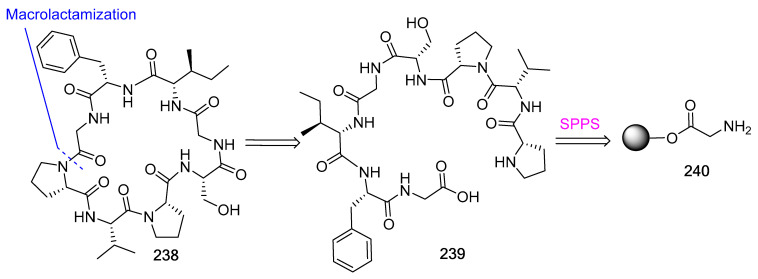
Retrosynthetic analysis of **238**, Cyclopurpuracin.

Synthesizing reverse peptides is an interesting area of medicinal chemistry. Reverse cyclic peptides contain the same sequence of amino acids and the same absolute stereochemistry but differ in the orientation of the amide bonds within the cyclic structure [225]. Although seemingly unimportant, the reverse configuration could induce a more pronounced (or diminished) helical dipole moment, potentially leading to different biological activities [226]. Yayat et al. [227] synthesized reversed cyclopurpuracin (**238**, Figure 63) after failing to make natural cyclopurpuracin. Starting with on-resin Gly **240**, the linear octapeptide was elaborated using standard SPPS protocol using a chlorotrityl chloride resin. The conditions for SPPS were optimized with HBTU/HOBt in DMF. Macrolactamization of **239** was achieved using HBTU and Hunig’s base (1%) under diluted conditions (1.25 mM), which afforded **238** in 20.8% yield. Subsequent 2D NMR studies (COSY, HMQC, HMBC, and NOESY) suggested the plausibility of two conformers in the sample. Natural cyclopurpuracin did not show these signals, and the reversed cyclopeptide showed a ratio of 54:46 of conformation A to B, respectively. NOESY and COSY aided the authors in drawing correlations between two conformers, as the 2D characterizations suggested, based on Δβγ values, the presence of a *cis*-Pro bond within conformer B. No biological studies were performed.

**Figure 64:**
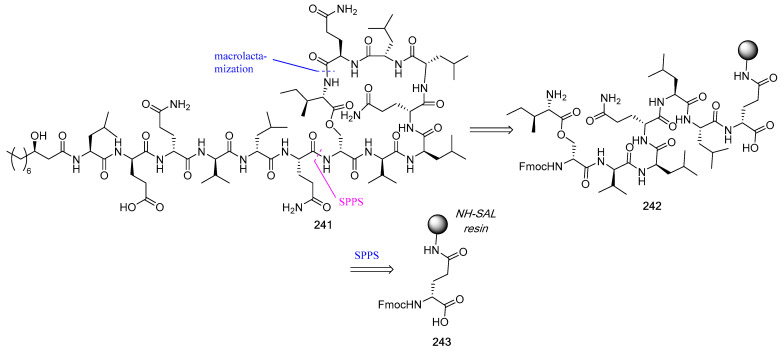
Retrosynthetic analysis of **241**.

MA026 (**241**, Figure 64) has implicated the biological activity of tight junction (TJ) opening activity [228] and was first isolated and characterized in 2002 by extracting from *Pseudomonas* sp. RtlB026. Total solution-phase synthesis of **241** and analysis of its anti-hepatitis C viral activity by binding to the TJ membrane protein claudin-1 was reported in 2013 by Shimura et al. [228]. Kanda et al. [229] also reported that natural **241** possessed TJ opening activity in 2017. However, the recent revision of the proposed structure was reported by the Hayashi group [230] after achieving the proposed structure through SPPS. Still, spectral data did not agree with the natural product. The revised synthesis was initiated with NH-SAL resin-loaded intermediate **243**. Using HATU/HOAt and Fmoc/SPPS protocol, the peptide backbone was elongated to linear precursor **242**. On-resin head-to-side-chain cyclization was achieved using DIPCI/HOBt between an embedded Gln residue and the terminal amine. The final peptide chain elongation was subsequently completed at the peptide bond adjacent to the ester linkage using DIPCI/HOBt or HATU/HOAt. Global deprotection and cleavage resulted in **241**. Following HPLC purification, the authors reported a 15% yield. The revised structure was tested for TJ opening, which showed similar activities to natural **241**.

**Figure 65:**
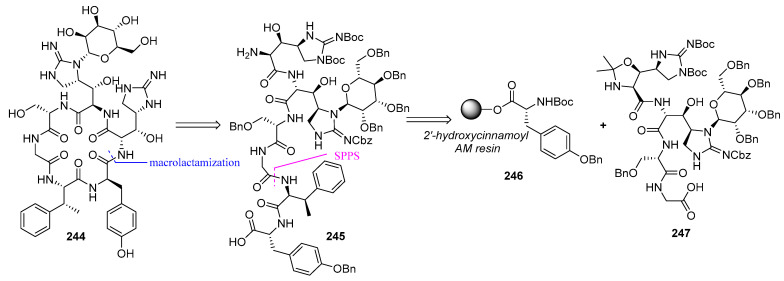
Retrosynthetic analysis of **244**.

Mannopeptimycins (MPPs) were isolated from *Streptomyces hygroscopicus* LL-AC98 by De Voe and Kunstmann in the 1950s [231]. Recently, their therapeutic properties have resurfaced and have shown promising activities against clinically resistant Gram-positive pathogens like methicillin-resistant *S. aureus* (MRSA) and vancomycin-resistant *Enterococci* (VRE) [232]. In 2002, the structures of MPPs were elucidated by Carter et al. [233], which represented the cyclic hexapeptides possessing altering d- and l-amino acid residues. Four of those residues are nonproteinogenic: d- and l-β-hydroxyenduracididines (βhEnd); l-β-methyl Phe (βMePhe); and d-Tyr. All MPPs contain an α-*N*-mannosylation at the βhEnd site. Wang et al. [234] devised a total synthesis of mannopeptimycin β (**244**, Figure 65) over 12–15 steps with reported yields similar to other strategies (6–11%). In the initial stages, a tripeptide building block **247** containing mannose and guanidine moieties was first synthesized in the solution phase over three steps. Using a 2′-hydroxycinnamoyl AM resin, the linear precursor **245** was synthesized in the presence of PyBOP and Hunig’s base, starting with on-resin intermediate **246**. Finally, the linear precursor was cyclized in a head-to-side-chain manner using β-hydroxyenduracididine ligation in a pyridine/HOAc 1:1 buffer (c = 2 mM), resulting in a cyclized *N*-acyl oxazolidine intermediate. Subsequent deprotection steps and acid-catalyzed hydrolysis of the *N*-acyl oxazolidine resulted in target **244**, purified by two consecutive RP-HPLCs. Characterizations were consistent with the previously reported literature. No biological studies were completed.

**Figure 66:**
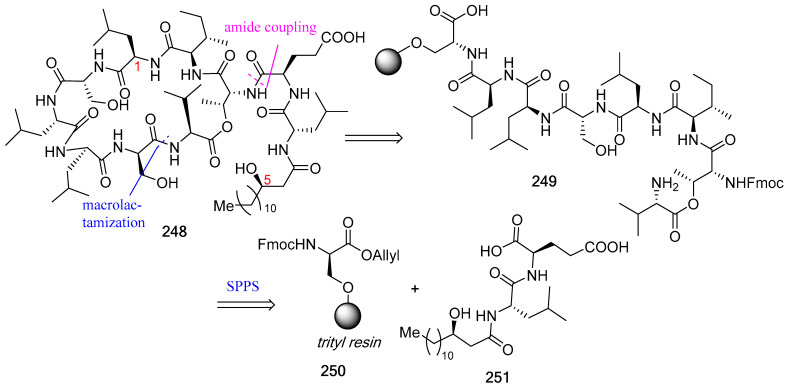
Retrosynthetic analysis of **248**.

Orfamide A (**248**, Figure 66) is categorized as a cyclic lipodepsipeptide that has been isolated from *Pseudomonas protegens* (Pf-5 or CHA0) [235,236]. Biological studies have characterized **248** as an insecticidal, antifungal, and algicidal agent [237,238,239,240]. Bando et al. [241] sought to synthesize the natural product, which features ten amino acids and a β-hydroxytetradecanoic acid (β-HTDA). The previous characterization established the structure of **248**, but three stereogenic centers had not been assigned. Ser intermediate **248** was loaded on a trityl resin for solid phase residue coupling, giving linear precursor **249**. Simultaneous allyl deprotection and macrolactamization were accomplished with on-resin head-to-side-chain coupling mediated by HBTU/HOBt (optimized yields = 35–44%). Several stereoisomers of **248** were established at this point by incorporating l- or d-Leu at positions 1 and 5 (Red, Figure 66). The final product was achieved by installment of the β-HTDA moiety (**251**, *S* or *R*, 3’ position) using HBTU/HOBt. Biological studies were conducted on *Trypanasoma brusei* and *C. reinhartii*, showing inhibited growth of both species at 6 μM.

**Figure 67:**
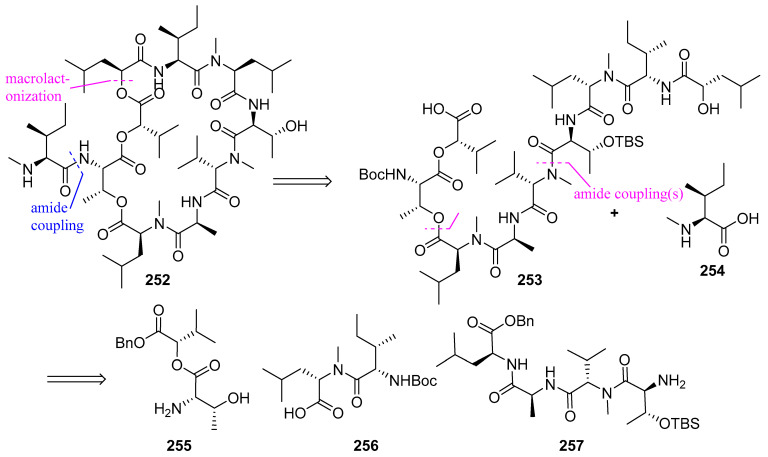
Retrosynthetic analysis of **252**.

Pleofungins are cyclic peptide antifungal agents first isolated in 2007 from *Phoma* sp. SANK 13899 by the Sankyo group [242]. They have been shown to inhibit fungal biosynthesis of inositol phosphorylceramide (IPC) via IPC synthase inhibition. Pleofungin A (**252**, Figure 67) has shown the most potent inhibitory activity against IPC synthase in *Aspergillus fumigatus* and *S. cerevisiae* (IC_50_ = 0.9 nM and 6.4 nM, respectively) [243]. Natural **252** is a 28-membered depsipeptide that forms a rectangular shape due to intramolecular hydrogen bonding. Kogen and Yokoyama [244] reported a total synthesis of **252**, starting by constructing three linear peptide fragments, **255**–**257**, and coupling them in the presence of EDCI and HOAt in the solution phase. The resulting linear precursor **253** was subject to head-to-side-chain macrolactonization. This was completed under Yamaguchi conditions at 4 mM with reported yields of ~71%. Once cyclized, an amide coupling of intermediate **254** was installed to complete the synthesis. After purification, the calculated overall yield was 6.3% under optimized conditions. Synthetic **252** was subject to comparative ^1^H and ^13^C NMR studies, and single crystals were also acquired and analyzed by SC-XRD for structural confirmation. No biological studies were reported.

**Figure 68:**
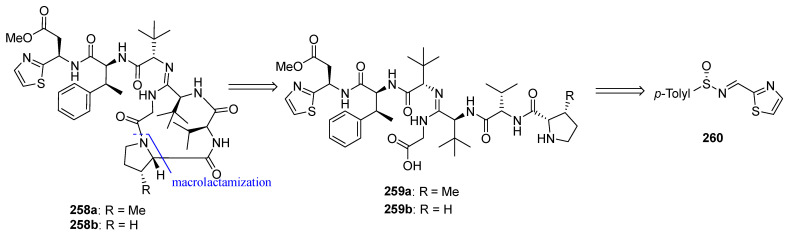
Retrosynthetic analysis of **258a** and **258b**.

Bottromycins are heptapeptides containing a cyclic tetrapeptide and a linear tripeptide attached to the ring by an amidine bond (Figure 68). The first bottromycin was discovered in 1957 by Waisvisz and co-workers and was found to have potent antibacterial activity, particularly against resistant strains [245]. In recent years, several peptides within the family were isolated from *Streptomyces* No. 3668-L2, and intense structural validation was also completed [246]. Yamada et al. [247] devised a reliable total synthesis of bottromycins A_2_ (**258a**) and B_2_ (**258b**) and evaluated their activities against Gram-positive bacteria, including antibiotic-resistant strains. Convergent solution-phase synthesis was utilized to generate the linear peptide precursors (**259a**,**b**), starting from intermediate **260**. Macrolactamization for each linear peptide was optimized by using EDCI as the coupling reagent, and cyclization was achieved through a tail-to-side-chain orientation. Both products were analyzed by ^1^H/^13^C NMR, IR, and HRMS and matched with their naturally sourced counterparts. Both **258a** and **258b** were evaluated against four different *S. aureus* strains, two of which were methicillin-resistant *S. aureus* (MRSA) and one vancomycin-resistant *E. faecalis* (VRE) strain using vancomycin as the positive control (all cases). Against all strains, both **258a** and **258b** showed moderate to low MICs. Product **258a** exhibited superior activity, with MICs ranging from 1 to 2 μg/mL, similar to vancomycin. In essence, **258a** MICs were the same as its natural counterpart. Product **258b** showed slightly lower activity, exhibiting MICs of 4 μg/mL against all strains.

Many natural cyclic peptides require a head-to-tail cyclization; the reported syntheses offer important implications when considering other forms of cyclization. We saw how challenging it was to acquire acceptable yields for head-to-tail cyclization. Often, this leads to the overuse of solvents and catalysts and is, thus, impractical from a pharmaceutical perspective. However, innovations evolved that can mitigate this, as seen with turn-inducers, solubilizing protective groups, and on-resin cyclizations. These highlight important innovations in the field of cyclic peptide synthesis and drug discovery, as these will facilitate the synthesis and manufacturing process for pharmaceutical development.

## 3. Side-Chain-to-Side-Chain

**Figure 69:**
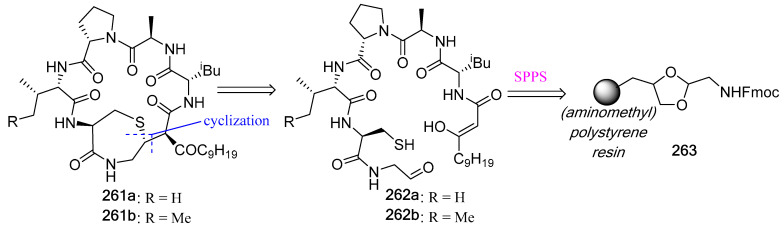
Retrosynthetic analysis of **261a** and **261b**.

Hansen et al. [248] developed a total synthesis strategy for synthesizing the cyclicpeptides mutanobactins A and B (**261a** and **261b**, respectively, Figure 69) as potential antifungal agents. Mutanobactins have been isolated from *S. mutans*, a common bacterium found in human oral biomes, and are presumed responsible for the etiology of dental caries. Mutanobactins A (**261a**) and B (**261b**) differ by one amino acid, with the former incorporating l-Val, whereas the latter features l-Ile. Both mutanobactins A and B contain a unique 1,4-thiazepan-5-one ring within the macrolactam, while mutanobactin D lacks the thiazepanone completely. Mutanobactin D was also synthesized, but the compound was inactive as an antifungal agent. Products **261a** and **261b** were synthesized the same way, using Fmoc-SPPS on an (aminomethyl)polystyrene resin starting with intermediate **263**. The macrocyclization step required linear intermediates **262a** and **262b** to be protected with the dioxolane group containing the decanoyl moiety. Cyclization was completed under acidic conditions (TFA), which involved deprotection and cyclization to complete the synthesis by way of side-chain-to-side-chain cyclization. Preparative HPLC was used to isolate the final product with an overall yield of 4–9%. An abiotic cyclic peptide was prepared to probe the necessity of the hydrophobic moiety (decanoyl) through a separate synthetic scheme. Biological studies were not provided.

**Figure 70:**
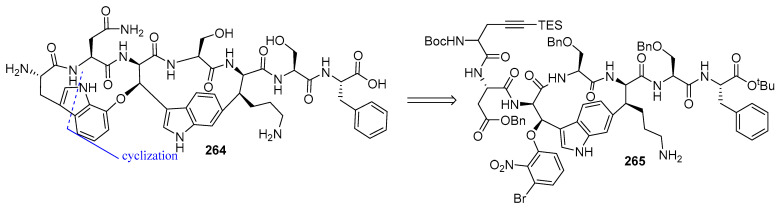
Retrosynthetic analysis of **264**.

Darobactin A (**264**, Figure 70) was first isolated from *Photorhabdus* bacteria and has shown promising antibiotic activities against Gram-negative bacteria [249]. Natural **264** has a unique strained bicyclic structure featuring two indole Trp heterocyclic rings within a 14- and 15-member ring system (Figure 70). The Baran group [250] designed a total synthesis of **264** which required at least two atroposelective steps for completion. The cyclization steps were crucial to their synthetic design and utilized two consecutive Larock side-chain-to-side-chain cyclizations (Figure 65 shows one example from intermediate **265**). The Larock reaction generates an indole from the Pd-catalyzed reaction between a disubstituted alkyne and an *o*-halogenated aniline (Br or I). After optimizing the conditions, Lin and co-workers were able to achieve the cyclizations atroposelectively using Pd(P^t^Bu_3_)_2_ with Gram-scale reported yields of 61% and 67% for the first and second cyclizations, respectively. Post-macrocyclization modifications were implemented to achieve the C-terminal via the coupling of a dipeptide. No biological studies were reported for product **264**.

**Figure 71:**
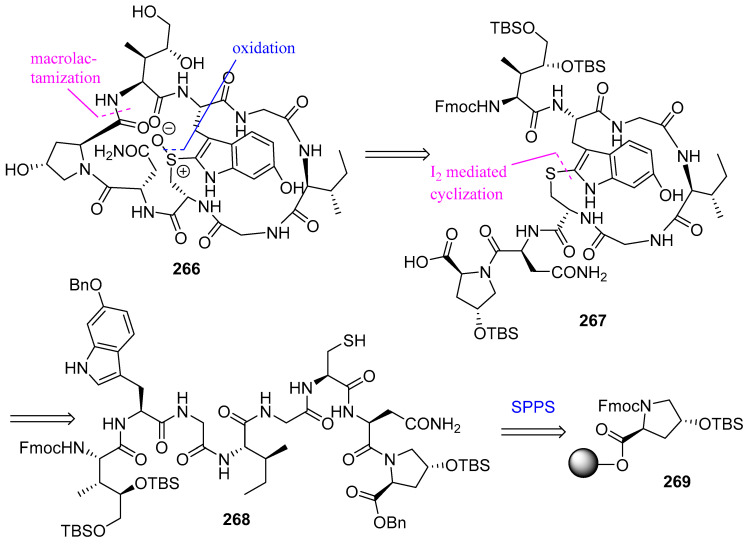
Retrosynthetic analysis of **266**.

The Sussmuth group [251] revisited the total synthesis of *a*-amanitin (**266**, Figure 71) utilizing SPPS and an iodine-mediated tryptathionine formation via side-chain-to-side-chain cyclization, followed by a head-to-tail macrolactamization. Amanitins are bicyclic octapeptides that feature the unique tryptathionine-derived 6-hydroxy-tryptathionine-(*R*)-sulfoxide moiety only found in death-cap toxins [252,253]. *α*-amanitin is a widely studied selective RNA polymerase II (RNAPII) inhibitor isolated from the death-cap mushroom *Amanita* phalloides [254] that has been synthesized previously through various routes [255]. The cytotoxic abilities of eukaryotic cells could facilitate strides toward new cancer therapies if a reliable synthetic method allows for high throughput SAR studies. The total synthesis of *a*-amanitin was used to test the synthetic utility of generating amanitin analogs. The synthesis began with **269** following Fmoc-SPPS of the linear precursor **268** using HBTU as the coupling agent. The side-chain-to-side-chain cyclization was carried out between the Trp and Cys residues in the presence of I_2_ (2 mg/mL) in DMF, resulting in **267**, a 40% overall yield from initial resin loading. A final cyclization, using macrolactamization conditions, was achieved between the amine head and Pro C-terminal using HATU to give a fully cyclized product. The bicyclic structure was subject to stereoselective oxidation of the tryptathionine moiety, resulting in **266** after global deprotection, confirmed by HPLC and NMR characterizations. A library of aminitins was synthesized using the same methods for SAR studies against RNA polymerase II.

**Figure 72:**
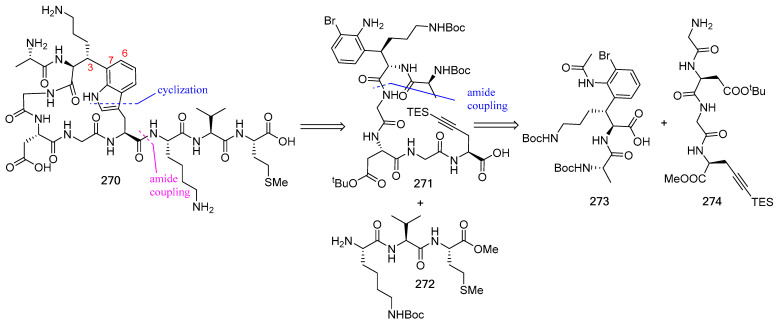
Retrosynthetic analysis of **270**.

Streptide (**270**, Figure 72) is a 20-membered cyclic peptide natural product isolated from *Streptococcus thermophilus* [256]. Its unusual structure contains a unique cross-link between the *β*-carbon of residue 2 Lys and C7 of residue 6 Trp. Because of limited availability, Isley et al. [257] sought a synthetic route to **270** to further study its properties. Two fragments were independently synthesized and combined at different stages. The first involved installing a functionalized aryl group at the β-position of a Lys analog using a diaryliodonium salt, resulting in **273**. It was subsequently coupled with a separate tetrapeptide **274** containing a protected alkyne functionality, resulting in intermediate **271**. Linear **271** underwent a side-chain-to-side-chain annulation using Pd(0)-mediated Larock indole conditions. Optimized conditions, affording the cyclic peptide precursor in 60% yield, utilized Pd(PtBu_3_)_2_ and Cy_2_NMe at 1 mM. Finally, the third fragment, tripeptide **272**, was coupled at the free amine of the cyclic precursor using HBTU. Purification using preparative HPLC methods isolated **270** after global deprotection. Natural and synthetic **270** were used in comparative NMR (1D and 2D) and HPLC studies, which confirmed the stereochemical properties of natural **270**. No biological analyses were performed.

**Figure 73:**
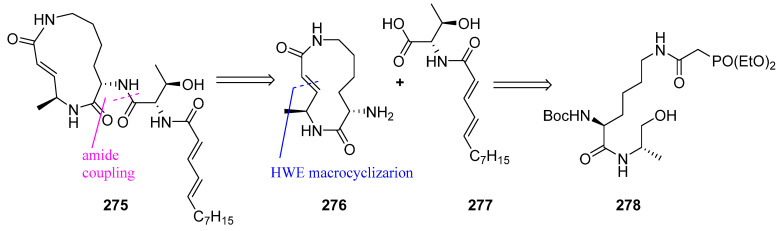
Retrosynthetic analysis of **275**.

From a *Photorhabdus luminescens* extract with silent megasynthetase [258], luminmycins A–D were identified as a new family of cyclic peptides, the luminmycins, which resemble the proteasome inhibitor glidobactin A [259,260]. Specifically, luminmycin A (**275**, Figure 73) has been shown to possess potent cytotoxic activity in vitro against both human colon (HCT-116, 91.8 nM) and pancreatic cancer (18 nM) cell lines [260]. Servatius, Stach, and Kazmaier [261] provided a total synthesis of **275** using a liquid-phase, convergent approach. Beginning with the construction of the peptide fragment, **278** was constructed in the solution phase and cyclized using a side-chain-to-side-chain intramolecular Horner–Wadsworth–Emmons (HWE) olefination, following a Dess–Martin primary alcohol oxidation. Under optimized conditions, cyclization was achieved in 50% yields, giving cyclized intermediate **276**. The final step involved synthesizing and appending fragment **277**, featuring a fatty acid chain and diene functionalities. This was installed under amide coupling conditions to yield **275** in 26%. No biological studies were performed.

**Figure 74:**
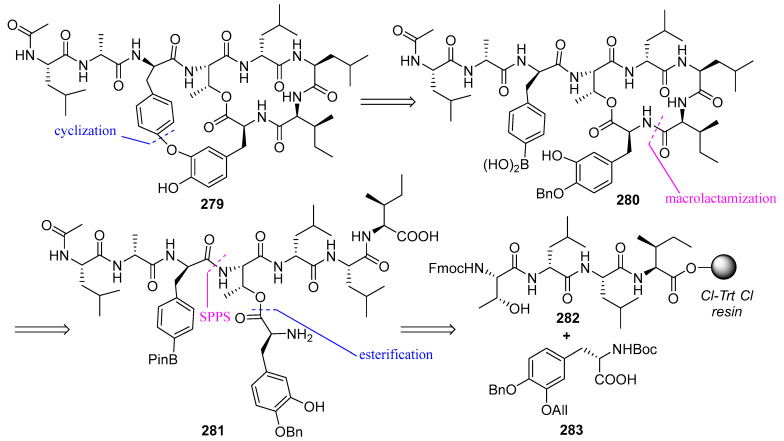
Retrosynthetic analysis of **279**.

Shabani and Hutton [262] reported the synthesis of bicyclic, natural peptide seongsanamide B (**279**, Figure 74) isolated from *Bacillus safensis*. After isolating six cyclic peptides (seongsanamides A-F), the family exhibited antiallergenic properties against bone marrow-derived mast cells (BMMCs) and inhibited β-hexosaminidase release. Natural **279** is bicyclic, containing a tetrapeptidyl ring and a biaryl ether ring system with an appended exocyclic dipeptide tail. The authors employed a traditional approach, using Fmoc-SPPS on a chlorotrityl resin, allowing for linear elaboration with HATU from synthetic intermediate **282**. Once the linear peptide was assembled, an esterification appended intermediate **283**, giving linear peptide **281**. The first cyclization occurred through head-to-tail lactamization, mediated by HATU/HOAt (0.8 mM), affording the monocyclic intermediate **257** in 51% yield. The second cyclization involved a side-chain-to-side-chain connection under Evans–Cham–Lam conditions with Cu(II), resulting in the bicyclic peptide **279** in 26% yield after deprotection. Overall, the authors reported a 3.6% yield over 16 steps. Comparative NMR characterizations (^1^H, ^13^C, COSY, NOESY, and ROESY) matched natural **279**. No biological studies were completed.

Although still challenging, cyclization via the side-chain-to-side-chain method often results in higher yields. This is most likely due to the closer proximity and requirement for more efficient coupling reactions than lactamization conditions. We reported examples where traditional chemical synthesis was applied on a macrostructural scale, giving rise to enhanced and extended knowledge of the corresponding conditions required for developing cyclic peptide pharmaceuticals.

## 4. Head-to-Side-Chain

**Figure 75:**
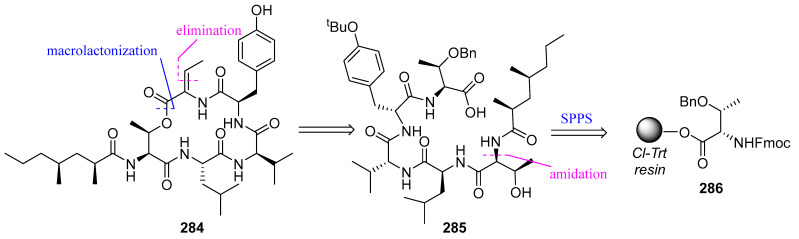
Retrosynthetic analysis of **284**.

Tumescenamide C (**284**, Figure 75), a cyclic pentadepsipeptide, was first isolated from a culture broth of *Streptomyces* sp. KUSC_F05 in 2012 by Kakeya [263]. The natural peptide exhibited antibacterial activity against Gram-positive *Streptomyces* sp., such as *Streptomyces coelicolor*, *Streptomyces lividans*, and the producing strain [263]. Apart from the five amino acid residues forming the ring structure, **284** also features an exocyclic dimethylhexane lipophilic tail. Takahashi et al. [264] designed a total synthesis for **284**, utilizing solid- and solution-phase chemistry. Intermediate **286** initiated the linear precursor elongation on a chlorotrityl resin in the presence of DIC/HOBt. The methylated hexane moiety was synthesized separately into dimethylhexanoic acid in the solution phase and appended through lactamization prior to cyclization. Intermediate **285** underwent head-to-tail macrolactonization under Shiina conditions, accomplishing the cyclization step, mediated by 2-methyl-6-nitrobenzoic anhydride (MNBA)/DMAP, resulting in a 25% yield (from resin loading). NMR (^1^H/^13^C) and HPLC retention times were in good agreement with that of natural **284**. Synthetic **284** was evaluated for antimicrobial activity against *Streptomyces lividans* TK23 using a disk diffusion assay, which revealed a MIC of 3 μg/disk.

**Figure 76:**
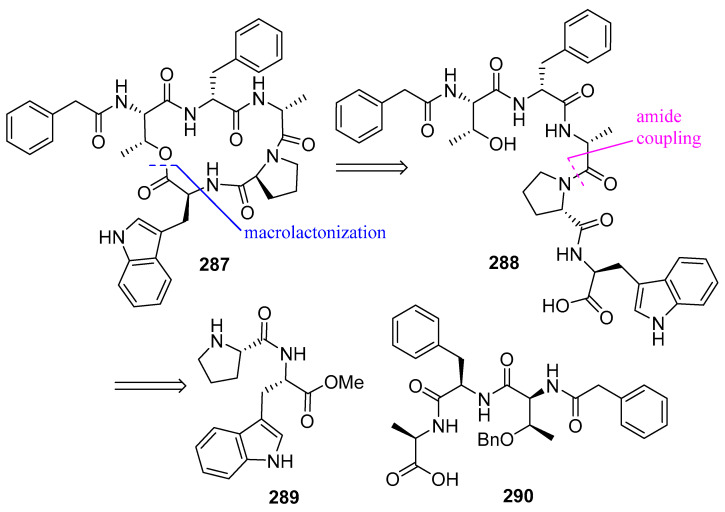
Retrosynthetic analysis of **287**.

The first total synthesis of chaiyaphumine A (**287**, Figure 76) was conducted by Gholap et al. [265] via a solution-phase convergent synthesis. Two small peptides, **289** and **290**, were independently elaborated in solution using HATU as the primary coupling agent. The two peptide fragments were coupled, again using HATU, resulting in **288**. Finally, following hydrolytic deprotection, head-to-side-chain macrolactonization was accomplished between Trp and Ser residues in the presence of propanephosphoric acid anhydride (T_3_P) under basic conditions (DIPEA). Optimizing these conditions allowed for a 12% cyclization yield of **287** following RP-HPLC purification. NMR studies and HRMS spectra matched that of the reported natural **287**.

**Figure 77:**
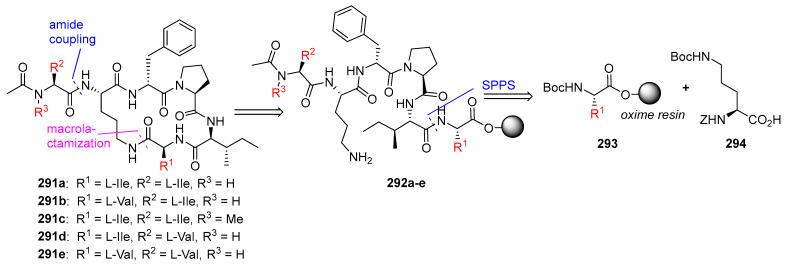
Retrosynthetic analysis of **291a**–**e**.

In 2010, five pseudacyclins, A–E (**291a**–**e**, Figure 77), were isolated and characterized by Pavlaskova et al. [266] when exploring the secondary metabolites of the pathogenic fungus *Pseudallescheria boydii sensu lato*. Biological analysis revealed in vitro evidence of psuedacyclins possessing immunosuppressive capabilities. Years later, Voyer and co-workers [267] established a total synthesis of **291a**–**e**, using an on-resin SPPS approach. All pseudacyclins contain the same amino acids (l-Pro, l-Phe, l-Val, l-Ile), an endocyclic l-Orn residue, and an exocyclic acylated Val or Ile. They differ only in the positional substitution of l-Val or l-Ile at positions 1 and 6 and an *N*-methyl alkylation at the exocyclic Ile (in the case of **291c**). The synthesis used an oxime resin, starting with intermediate **293**. The orthogonally protected l-Orn, **294**, was incorporated during this process, allowing for the elongation of each linear precursor (**292a**–**e**) using HCTU under basic conditions. Head-to-side-chain macrolactamization was promoted concomitantly by an on-resin cyclization/cleavage using DIPEA and AcOH, affording the cyclic precursors in reported 70–72% yields. The acylated tail amino acid was coupled in the final step using ECI/6-Cl-HOBt, affording product **291a**–**e**. Overall yields of the final products were 22–28%. ES-MS and HLPC retention times, along with NMR, were compared and matched to the literature characterizations. No biological studies were performed.

**Figure 78:**
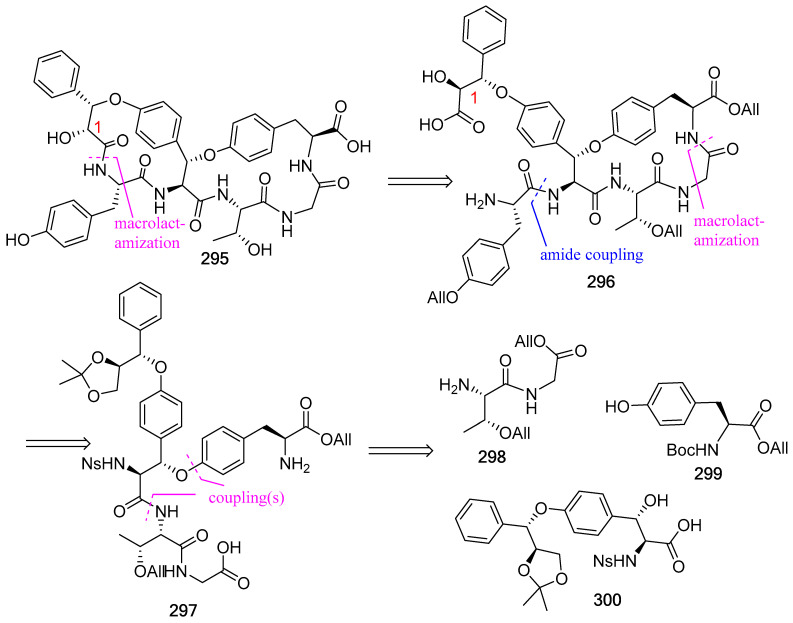
Retrosynthetic analysis of **295**.

Asperipin-2a (**295**, Figure 78) is a bicyclic RiPP isolated from *Aspergillus flavus* in 2016 [268]. The uniquely structured peptide had not been studied biologically, prompting Shabani et al. [269] to report the first total synthesis of **295**. The synthesis started with key intermediate **300**, and the cyclization precursor **297** was divergently synthesized using fragments **298** and **299**. The first cyclization occurred through a head-to-side-chain macrolactamization, using HATU/HOAt (0.6 mM), yielding the cyclic intermediate **296** in 55% isolation. Further functionalization allowed for the second cyclization, performed in a head-to-tail directionality, again using HATU/HOAt, resulting in a bicyclic intermediate in 64% yields. Finally, the authors employed a Lattrell–Dax inversion, targeting the C1 secondary alcohol followed by global deprotection to give **295**. Comparative chiral HPLC, assisted by NMR (1D and 2D), allowed for complete structural elucidation of synthetic **295** as being the same as its naturally sourced product, asperipin-2a. No biological evaluations were reported.

As we have seen, synthetic visions needing a head-to-side-chain cyclization require the presence of a carbonyl and a nucleophilic or redox-susceptible group. Concentration is still a major consideration. Some conditions were analogous to those seen in head-to-tail cyclizations. Others, however, curtailed conventional organic synthesis to larger, more sterically unpredictable compounds. Thus, these powerful tools should be considered in a drug development setting.

## 5. Tail-to-Side-Chain

**Figure 79:**
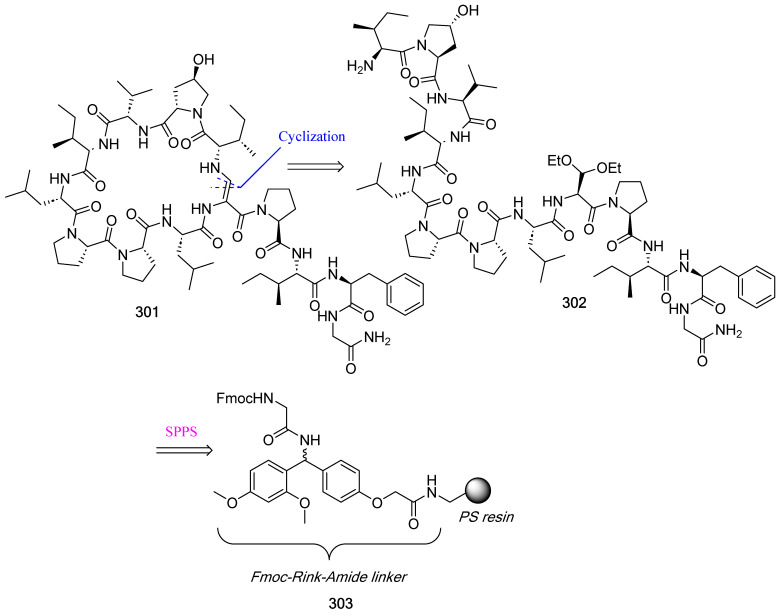
Retrosynthetic analysis of **301**.

Zhang et al. [270] described a total synthesis of callyaerin A (**301**, Figure 79), a Pro-rich cyclic peptide, isolated from the marine sponge *Callyspongia aerizusa*, a potent inhibitor of *M. tuberculosis* (MIC_100_ of 6.0 μM) [271]. The peptide features a unique (*Z*)-2,3-diaminoacrylamide (DAA) moiety within the ring structure that houses nine endocyclic amino acid residues, while four are exocyclic. The authors envisioned a cyclization resulting in the formation of the DAA moiety through an imino-mediated cyclization. Still, their attempts to directly oxidize Ser or Cys residues to an aldehyde were unsuccessful. Alternatively, an acetal-protected Fmoc-formylglycine was installed and utilized as the precursor to a DAA-forming cyclization. Linear elaboration of the peptide was constructed using SPPS on a Fmoc–Rink amide aminomethyl polystyrene resin in the presence of HATU, starting with intermediate **303**. After resin cleavage, the linear precursor **302** was obtained in 87% yield and used directly for solution-phase cyclization. After a tail-to-side-chain imino-mediated cyclization, product **301** was obtained in 81% yield, using 1% (*v*/*v*) formic acid in MeCN at 0.75 mM, following HPLC purification. ^1^H and ^13^C NMR studies were in good agreement with natural **301**. Evaluation against *M. tuberculosis* showed an MIC_100_ = 32 μM. Further temperature-dependent NMR studies exemplified the conformation rigidity of **301** due to the DAA moiety.

## 6. Discussion

Recent advancements, including the incorporation of turn-inducing motifs, solubilizing protective groups, and on-resin cyclization techniques, have markedly improved the efficiency and scalability of these processes. Among all strategies, head-to-tail cyclization heavily relied on labor-intensive solution-phase techniques characterized by high solvent consumption and limited control over stereochemistry. In contrast, modern innovations, such as solid-phase peptide synthesis (SPPS), have facilitated a streamlined process by providing controlled environments for cyclization while reducing the risk of byproduct formation [272].

Introducing turn-inducing motifs has substantially transformed the field, promoting cyclization at lower concentrations and diminishing excess reagent requirements. These advancements signify a substantial improvement over the historically inefficient and resource-intensive methods. Furthermore, head-to-tail cyclization directly impacts the therapeutic potential of cyclic peptides by stabilizing their structures, reducing enzymatic degradation, and enhancing bioavailability. Numerous approved cyclic peptide therapeutics, including cyclosporine and vancomycin, depend on head-to-tail cyclization to exhibit their biological activity against various diseases, including infections, cancers, and autoimmune disorders. Improved synthesis methodologies are essential for broadening the therapeutic applications of cyclic peptides, as they permit the production of more diverse and complex structures tailored to specific biological targets [273].

Several emerging trends are promising avenues for addressing the challenges inherent in peptide synthesis [274]. Developing novel turn-inducing motifs and solubilizing protective groups has significantly improved cyclization efficiency while minimizing racemization. Additionally, strides in solid-phase peptide synthesis (SPPS) now facilitate on-resin cyclizations, streamlining workflows, reducing waste, and enhancing scalability. Integrating automated peptide synthesizers further presents the potential to optimize head-to-tail cyclization processes and minimize variability. Moreover, computational tools, such as modeling and molecular dynamics simulations, are increasingly aiding researchers in predicting cyclization efficiency and identifying optimal reaction conditions [275].

Despite these advancements in cyclization reactions, several notable gaps persist that require attention. A deeper mechanistic understanding is crucial, as it may lead to the development of more efficient catalysts and optimized reaction conditions. Furthermore, scalability remains a significant challenge, with many existing methodologies proving difficult to implement on an industrial scale, underscoring the need for robust and reproducible processes. Enhancing the diversity of turn-inducing motifs specific to various peptide sequences could further augment cyclization efficiency. Lastly, adopting green chemistry practices, including solvent-free reactions and using recyclable catalysts, is essential for promoting sustainability in pharmaceutical development.

## 7. Conclusions and Future Perspectives

Within the last five years, we witnessed the development of strategic syntheses to unlock the increasing structural complexity of cyclic peptides. The cyclization strategies covered included head-to-tail, tail-to-side-chain, head-to-side-chain, and side-chain-to-side-chain by various covalent linkages. These distinguished accounts of research significantly contribute to developing, evaluating, and understanding cyclic peptides in nature as promising pharmaceuticals in nearly all facets of medicine. Such research has allowed chemists to develop these natural products at higher-than-naturally occurring quantities, allowing biologists to characterize and evaluate their therapeutic potential.

Synthesizing cyclic peptides, therefore, lays the groundwork for scientists in several ways. First, the synthetic intervention of a natural cyclic peptide confirms the stereochemical assignments and generates spectroscopic standards for future synthetic works. The synthetic routes and conditions for generating natural cyclic peptides in reasonable quantities facilitate more biological studies, the discovery of different therapeutic targets, and enhancement with SAR studies. These natural products can be synthesized and further derivatized to contain differing amino acid sequences, noncanonical elements, or exocyclic functional groups to obtain more specific and improved biological activity.

The latter is a subcategory in studying natural cyclic peptides. Although many cyclic peptides possess exceptional binding specificity, other factors, such as cell permeability, proteolytic stability, and absorption, play a large role in effective biological response. Therefore, there is a call for chemists, biologists, and computational scientists to assist in delivering effective therapies. This task would, therefore, pose exceptional difficulty without first establishing synthetic routes and structural confirmation. In near-future directions, the powerful methods for synthesizing and testing large numbers of cyclic peptides may soon combine with computational and artificial intelligence for sampling a larger chemical space to develop highly potent cyclic peptide pharmaceuticals.
